# Genetically Encoded Aminocoumarin Lysine for Optical Control of Protein–Nucleotide Interactions in Zebrafish Embryos

**DOI:** 10.1021/acschembio.3c00028

**Published:** 2023-06-05

**Authors:** Wes Brown, Joshua Wesalo, Subhas Samanta, Ji Luo, Steven E. Caldwell, Michael Tsang, Alexander Deiters

**Affiliations:** † Department of Chemistry, University of Pittsburgh, Pittsburgh, Pennsylvania 15260, United States; ‡ Department of Developmental Biology, University of Pittsburgh, Pittsburgh, Pennsylvania 15260, United States

## Abstract

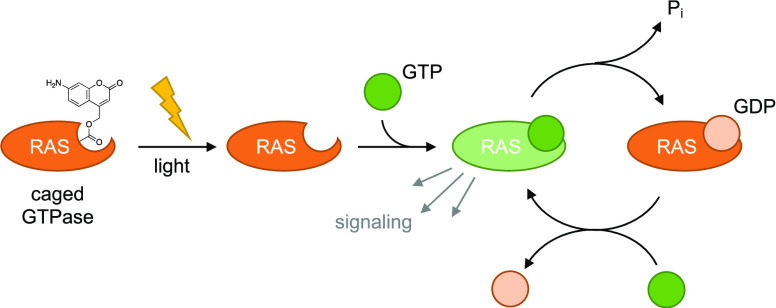

The strategic placement of unnatural amino acids into the active site of kinases and phosphatases has allowed for the generation of photocaged signaling proteins that offer spatiotemporal control over activation of these pathways through precise light exposure. However, deploying this technology to study cell signaling in the context of embryo development has been limited. The promise of optical control is especially useful in the early stages of an embryo where development is driven by tightly orchestrated signaling events. Here, we demonstrate light-induced activation of Protein Kinase A and a RASopathy mutant of NRAS in the zebrafish embryo using a new light-activated amino acid. We applied this approach to gain insight into the roles of these proteins in gastrulation and heart development and forge a path for further investigation of RASopathy mutant proteins in animals.

## Introduction

Embryo development involves an ensemble of tightly regulated cell signaling events that direct cell fate, movement, and proliferation.^1−3^ Many of these events are driven by phosphorylation pathways that utilize nucleotides as cofactors for the transfer of phosphate groups or to promote conformational changes, such as kinases and GTPases, respectively. Many enzymes in these families have an essential lysine in the nucleotide-binding pocket to correctly orient the substrate for catalysis.^4^ We have taken advantage of this to optically control the activity of kinases by replacing said lysine with a photocaged analogue using genetic code expansion.^5,6^ The photocaged enzyme can be spatially and temporally controlled, which has been used to study pathway kinetics, crosstalk, and adaptation.^7,8^ Genetic code expansion had recently been established in zebrafish embryos^9−11^ and has been applied to the photocaging of MEK1.^9^ This technology can provide insight into developmental signaling because signaling events in the embryo context are spatiotemporally constrained.^12^ Here, we developed a new photocaged lysine unnatural amino acid (UAA) and genetically encoded it in mammalian cells and zebrafish. Furthermore, two new signaling enzymes were placed under optical control in zebrafish embryos, Protein Kinase A (PKA) and the GTPase NRAS.

Mutations in the RAS/MAPK signaling pathway are the underlying cause of RASopathies, a family of congenital birth defects.^13^ While all mutations occur in a single pathway that terminates in the activation of ERK, a broad spectrum of clinical presentations are observed that can include craniofacial defects, neurocognitive impairment, skin abnormalities, and heart defects.^14−17^ Many clinical features overlap among these diseases, but some do not, and although the reason for this is unknown, it is likely due to crosstalk that certain nodes in the RAS/MAPK pathway have with other pathways.^18−20^ There is a wide swath of phenotypic effects on the embryo because of the involvement of RAS/MAPK signaling in many developmental processes during embryogenesis. However, it is difficult to isolate the impacts a given RASopathy mutant has on individual developmental processes like gastrulation, tissue differentiation, or organ development because of the spatial and temporal ubiquity of RAS/MAPK signaling in the embryo. NRAS mutants can cause Noonan syndrome, a class of RASopathies and the second most common syndromic cause of heart defects, and the G60E mutant showed a potent induction of developmental defects in zebrafish embryos. Zebrafish are an excellent model for studying development because they are completely transparent during early embryogenesis, making them amenable to in vivo imaging and also optical manipulation. We expanded the scope of photocaged proteins to study zebrafish embryo development and to pave the way for further detailed studies of the impact of these signaling components on specific developmental events, especially for the study of RASopathy mutant proteins.

## Results and Discussion

**Figure 1:**
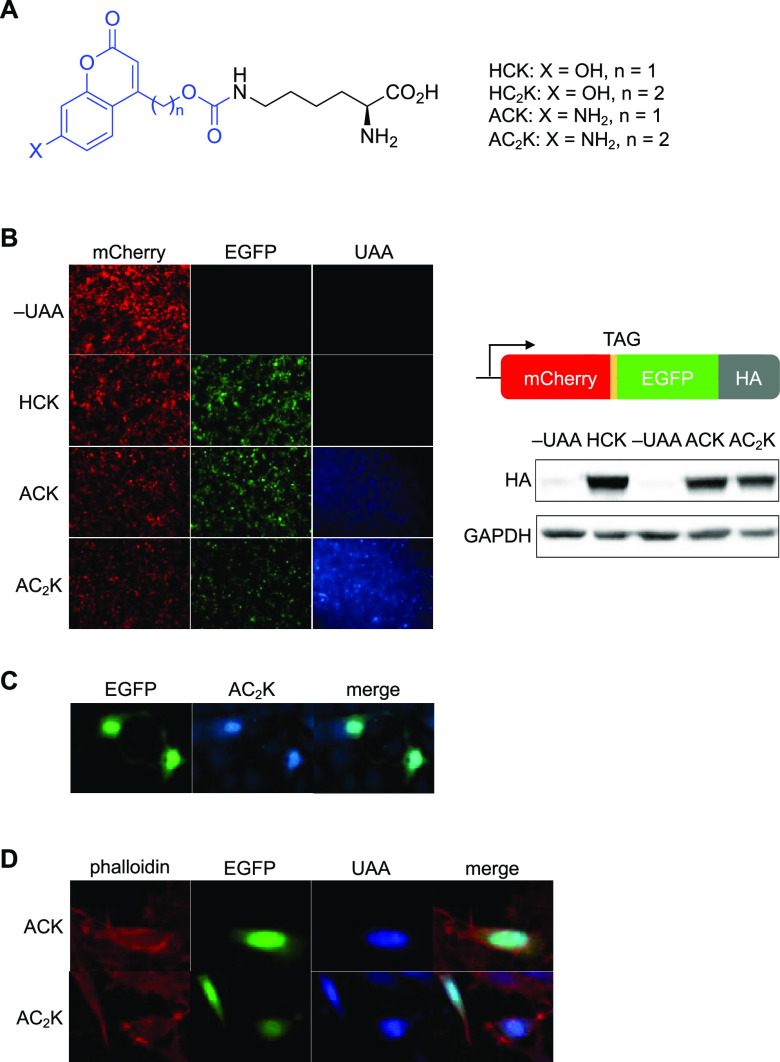
Incorporation of ACK and AC_2_K into proteins in mammalian cells. (A) Chemical structures of HCK, HC_2_K, ACK, and AC_2_K. The photolabile group/fluorophore is colored blue. (B) Confirmation of ACK and AC_2_K incorporation into a reporter construct through fluorescence imaging in HEK293T cells (10× magnification) and western blot. (C) Expression of NLS-EGFP-AC_2_K in live NIH 3T3 cells (20× magnification). (D) Fixed HeLa cells expressing NLS-EGFP-ACK (top row, 40×) and NLS-EGFP-AC2K (bottom row, 63×). Cells are counterstained with rhodamine–phalloidin.

To attain spatial and temporal control over proteins involved in RASopathy, we designed a sensitive, rapid-acting, and generally applicable photocaged UAA based on published reports of aminocoumarins. We previously reported 7-hydroxycoumarin-lysine (HCK)^21^ and used it to control several proteins in cells and in zebrafish (Figure 1A).^22−24^ We also found that HCK, and its homologue HC_2_K, act as genetically encoded fluorescent probes that can be observed in live cells. For the 7-hydroxycoumarin scaffold, however, only the phenolate form is absorptive. The phenol’s p*K*_a_ ranges from 7.5^25^ to 7.8.^26,27^ The majority of the UAA is nonfunctional at physiologic pH (7.4). With a reported analogue of HCK, absorptivity is less than 35% of maximal at pH 7.4 and tapers to less than 10% at pH 7 (and fluorescence intensity for 7-hydroxycoumarin decreases similarly with decreasing pH),^28,29^ suggesting that this motif is inefficient for use in neutral or mildly acidic subcellular compartments (e.g., endosomes at pH 5.5–6.5,^30^ or the Golgi apparatus at pH 6.1–6.6^31^), as well as acidic patches on proteins.^26^ Thus, we considered whether we could modify the scaffold to improve function at physiologic pH and below. As secondary goals, we also sought to improve on 7-hydroxycoumarin’s quantum yield for decaging (0.025)^32^ and for fluorescence (0.21),^33^ as well as red-shifting its λ_max_ (324 nm) toward less toxic, more practical wavelengths.

We selected aminocoumarin lysine (ACK) (Figure 1A) for our studies, based on reported results using the 7-aminocoumarin as a superior caging group^33^ and fluorophore,^29^ and synthesized it in 6 steps (SI Scheme S1). Additionally, we synthesized its homologue AC_2_K, which has a second methylene unit inserted between the lysine and the fluorophore to prevent photocleavage, in 8 steps (SI Scheme S2). We measured the extinction coefficient (ϵ) for ACK at 2.1-fold higher than that of HCK (Figure S1).^28^ Further, λ_max_ was bathochromically shifted to 348 nm for ACK (up from 330 nm for HCK). The quantum yield (ϕ) for decaging was measured as 0.04,^33^ nearly twice that of 7-hydroxycoumarin (0.025).^32^ Taken together, these properties make 7-aminocoumarin more practical for excitation with visible light compared to hydroxy analogues. Overall, considering the increased quantum yield and absorptivity, we expected 7-aminocoumarins to decage 3.4-fold more efficiently compared to 7-hydroxycoumarins.

To evaluate decaging at various biological pH levels, we next demonstrated in an LC-MS assay that after 405 nm light exposure, ACK decaged across a pH range of 5–8. HCK, by contrast, exhibited decreasing decaging efficiency with decreasing pH (Figure S2). Decaging was negligible below pH 6 and was still incomplete at neutral pH. We further studied decaging at pH 7.4 with increasing light exposure. When irradiated at 365 nm, the amount of lysine released from ACK was consistently higher (37–100%) than the amount released from HCK (Figure S3A). At 405 nm, which is commonly available on microscopes, the difference was even starker, with 4- to 8-fold greater lysine release from ACK compared to HCK at all tested conditions (Figure S3B). With 405 nm irradiation, lysine release from HCK was only 25% complete after an extended 120 s irradiation, whereas >50% release was attained within 10 s of irradiation using ACK, and the amount released approached 90% in this LC-MS assay after 120 s of irradiation (Figure S3B).

We first tested the incorporation of ACK and AC_2_K into sfGFP in *Escherichia coli*. ACK and HCK share many structural features, and thus we suspected that the evolved aminoacyl tRNA synthetase for HCK (HCKRS) would recognize ACK as well. In addition, HCKRS had been shown to tolerate other variations of HCK, including analogues with an extra methylene unit between the carbamate and coumarin ring and with a bromine at the 6-position of the coumarin ring.^21^ To further validate this assumption, we performed docking experiments with HCK or ACK into the HCKRS binding pocket (Figure S4) and found that both coumarin groups reside in nearly identical orientation. Both the coumaryl hydroxy (HCK) and amino (ACK) groups are positioned for hydrogen bonding with D373. We co-transformed cells with a plasmid encoding HCKRS and a plasmid encoding the reporter gene and PylT, and observed UAA-dependent expression (1 mM UAA in media) of sfGFP-Y151ACK and sfGFP-Y151AC_2_K with yields of 2.4 and 0.8 mg/L culture, respectively (Figure S5A). Successful incorporation was confirmed by whole-protein mass spectrometry (Figure S5B,C). Next, we tested incorporation of ACK and AC_2_K into a fluorescent reporter protein in HEK293T cells where mCherry fluorescence acts as a transfection control and EGFP fluorescence indicates incorporation efficiency (Figure 1B). The addition of ACK or AC_2_K to media for cells transfected with plasmid encoding HCKRS and PylT, along with the reporter plasmid resulted in high expression of EGFP. This was further confirmed by western blot probing for the C-terminal HA tag, showing efficient amber stop codon suppression and expression of full-length protein. Additionally, after washing the cells to remove excess UAAs, blue fluorescence (using a standard DAPI filter) was used to evaluate these coumarin residues as genetically encoded fluorophores. The signal for HCK was negligible (despite higher incorporation efficiency for HCK, which was previously expressed with yields of 8.0 mg/L culture^21^) at the conditions used to image ACK and AC_2_K. AC_2_K > ACK both gave strong fluorescent signals in all GFP-positive cells. These results suggest that as predicted from their greater absorptivity, quantum yield, red-shifted absorbance spectrum, and lack of pH dependence, aminocoumarins are brighter fluorophores in cells at physiologic pH than hydroxycoumarins.

We next tested ACK and AC_2_K’s fluorescence in other proteins and cell types. First, we genetically encoded both UAAs in EGFP conjugated to a nuclear localization sequence (NLS-KTAG-EGFP;^21^Figure 1C) in live NIH 3T3 cells and observed complete colocalization of AC_2_K with the GFP signal. We next tested ACK and AC_2_K in fixed HeLa cells with NLS-KTAG-EGFP (counterstained with rhodamine–phalloidin; Figure 1D). We observed a strong fluorescence signal for both UAAs that colocalized completely with EGFP. These results demonstrate that both UAAs can be used as reporters of protein localization in various subcellular compartments and that they function in both live cells (Figure 1B,C) and fixed specimens (Figure 1D). Further, their red-shifted absorbance spectrum relative to hydroxycoumarins allowed us to use both a mercury arc lamp (365 nm; Figure 1B,C) and a more red-shifted light-emitting diode (LED) (388 ± 8 nm) light source (Figure 1D) for effective illumination. These UAAs are among the smallest fluorescent tags for proteins and are unlikely to perturb protein function (e.g., nuclear localization as observed here) unless a critical site is selected for mutagenesis.

**Figure 2:**
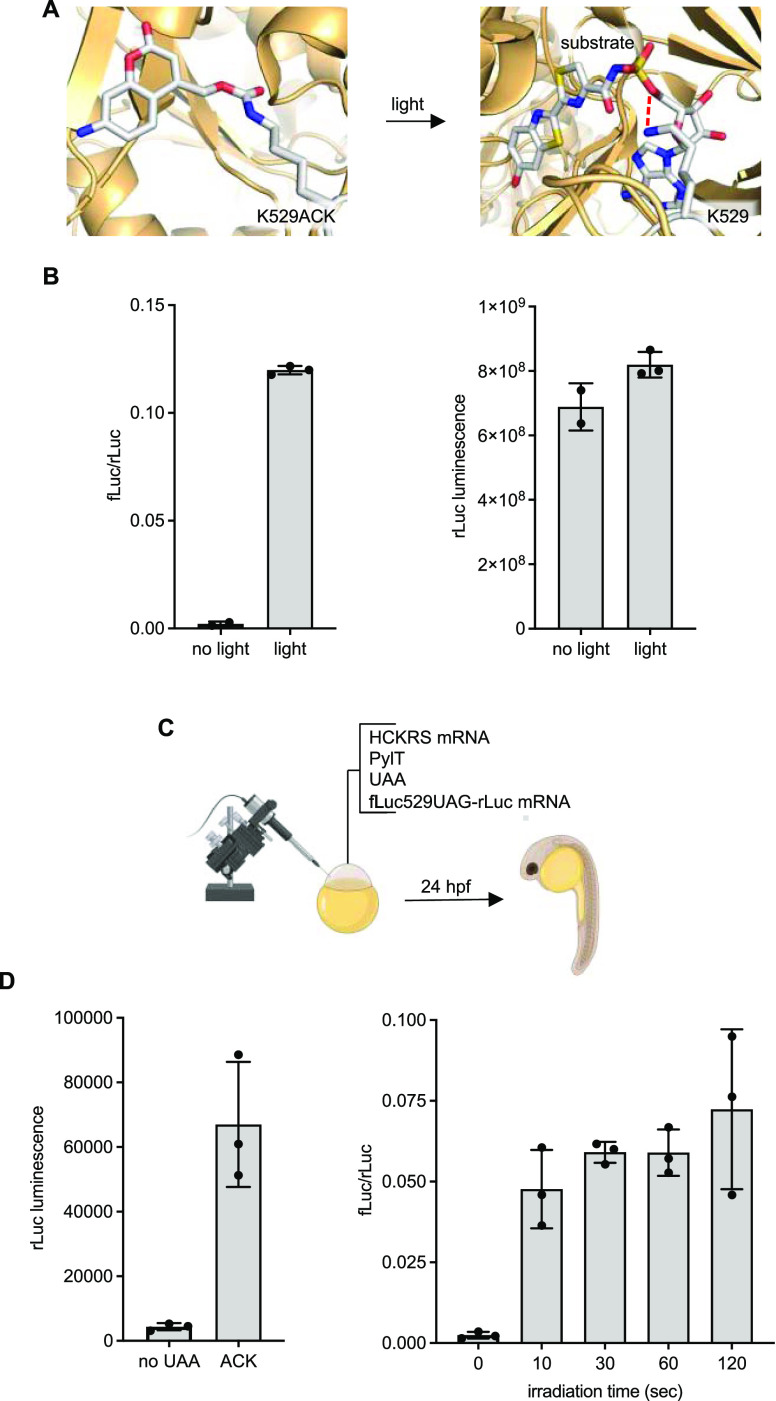
Incorporation of ACK into luciferase. (A) Structural representation of caging the fLuc active site with ACK (models generated from PDB: 4G36). Red lines indicate hydrogen bonding between K529 and luciferin–adenylate (here, a sulfate analogue as the substrate). (B) Incorporation of ACK into a dual-luciferase reporter and photoactivation of fLuc activity with 405 nm light in mammalian cells. Bars represent mean and error bars represent standard deviations of biological duplicates (no light) or triplicates (light). (C) Incorporation of ACK into a dual-luciferase reporter in zebrafish embryos is readily accomplished through injection of the mRNAs, tRNA, and UAA. (D) Luciferase incorporation and photoactivation assays. Embryos were irradiated with a 405 nm LED for the indicated amount of time. Bars represent mean, and error bars represent standard deviation from three pooled lysates of 4 embryos each.

To assess decaging of ACK with 405 nm light in vivo, ACK was incorporated into a dual-luciferase reporter fLuc K529TAG-rLuc. The amber stop codon is in place of a critical lysine residue essential for fLuc activity that orients the substrate and stabilizes the adenylated intermediate during catalysis through a hydrogen-bonding interaction with the phosphate oxygen (Figure 2A).^34^ The fused rLuc acts as a reporter for UAA incorporation. HEK293T cells expressing the caged reporter in the presence of UAA (1 mM) showed high rLuc activity, suggesting efficient incorporation of ACK, but had no fLuc activity (normalized to rLuc values to account for cell-to-cell differences in transfection), confirming inhibition of catalytic function in the absence of light (Figure 2B). After irradiation with 405 nm light, a 56-fold increase in fLuc activity was immediately observed, indicating decaging of ACK and restoration of the native active site. In comparison, the rLuc values did not change with irradiation, suggesting no photodegradation of the enzyme. The high rLuc activity coupled with the excellent optical off-to-on switching of fLuc activity suggests that ACK incorporates very efficiently into proteins, is able to disrupt key interactions in the active site of an enzyme, and is readily removed through a short exposure to 405 nm light.

Next, we adapted this methodology for incorporation in zebrafish embryos. The HCKRS and luciferase reporter genes were cloned into the pCS2 vector, commonly used as a template for in vitro transcription to generate mRNA (Figure S6). For generating PylT, a T7 promotor was appended to the 5′ of DNA encoding PylT by PCR and in vitro transcription was performed. mRNAs for HCKRS and the luciferase reporter were injected along with PylT and ACK (5 pmol total) into the one-cell stage embryo (Figure 2C). At 24 h post-fertilization (hpf), embryos were irradiated with 405 nm light and collected for lysis and luciferase assays. Incorporation of ACK was high, as indicated by rLuc values, and specific with complete orthogonality to the endogenous protein biosynthetic machinery in zebrafish, as indicated by low background in the absence of the UAA. Activation of fLuc was already observed after a brief 10 s irradiation and plateaued after a brief 2 min exposure to 405 nm light (extended irradiation of 5 min led to photodegradation of the enzyme), with no background activity before irradiation (Figure 2D).

To further validate optically controlling enzyme function beyond luciferase, we caged a critical lysine in Cre recombinase, K201TAG,^35^ which hydrogen bonds with the DNA backbone phosphate and assists the 5′-O as a leaving group to facilitate the cleavage reaction. These experiments were performed in a Cre recombinase reporter transgenic fish line. Exposure to light at 6 hpf for 2 min resulted in activation of Cre recombinase activity and switching from the expression of EGFP to mCherry (Figure S7), while embryos kept in the dark only expressed EGFP.

Taken together, these results confirm that ACK can be used to control enzyme function in zebrafish embryos through irradiation at 405 nm. Successfully blocking lysine–phosphate interactions in both luciferase and Cre recombinase sets the stage for optical control of other enzymes that use (oligo)nucleotide substrates or cofactors.

**Figure 3:**
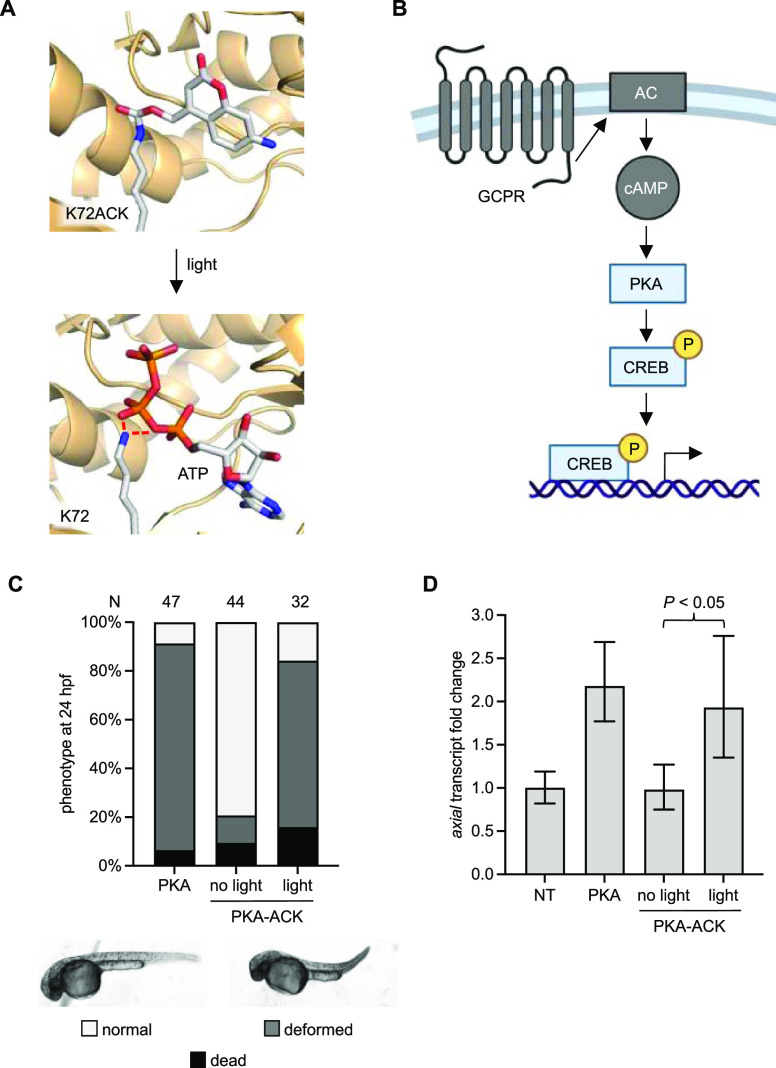
Photoactivation of Protein Kinase A (PKA). (A) Structural representation of the caged PKA nucleotide-binding pocket before and after light exposure. Red lines indicate hydrogen bonds (models generated from PDB: 4WB5). (B) The caPKA mutant is insulated from upstream-coupled protein receptor (GPCR) and adenylate cyclase (AC) interactions. (C) Embryos expressing PKA-ACK were irradiated at 4 hpf and imaged at 24 hpf. *N* = number of embryos. (D) Reverse transcription-quantitative polymerase chain reaction (RT-qPCR) measurement of the *axial* transcript. Bars represent mean, and error bars represent standard deviation from three independent pools of 50 embryos. An unpaired two-tailed Student’s *t*-test was performed between the two samples indicated. NT = nontreated embryos.

PKA was the first kinase to be crystallized to reveal the general structure of many protein kinases,^36^ including a critical lysine residue that interacts with the nucleotide triphosphate to orient it for catalysis. We took advantage of this knowledge to rationally design optical control of PKA. At least 197 different proteins are substrates for PKA phosphorylation implicating that PKA has many biological roles.^37^ Its role during development is less understood, but some studies have shown important interactions with sonic hedgehog signaling and activin signaling during development that lead to defects if disrupted.^38,39^ A lysine at site 72 interacts with ATP, so we inferred that replacement of K72 with ACK would disrupt this interaction (Figure 3A).^40^ PKA is activated by cyclic AMP, which leads to dissociation of the inhibitory domain from the kinase domain. We used a constitutively active mutant of PKA (caPKA, referred to as PKA in this study) to bypass the need for upstream signal activation and cAMP production for full PKA activity (Figure 3B).^41^ Injection of mRNA for PKA (Figure S8) led to a distinct body axis defect in embryos as imaged at 24 hpf (Figures 3C and S9).^42^

The majority of embryos expressing the caged caPKA (PKA-ACK) were phenotypically normal, but irradiation with 405 nm light at 4 hpf (before gastrulation) resulted in most having the same body axis defect. Hyperactivation of PKA has been shown to increase *axial* transcripts in conjunction with activin signaling in the blastula stage zebrafish embryos, a marker for mesoderm induction.^38^ However, at later somite stages, PKA was shown to decrease *axial* transcript levels, linked to a suppression of sonic hedgehog signaling.^39^ We performed RT-qPCR analysis on zebrafish embryo lysates collected at 6 hpf (early gastrula) after activation of PKA-ACK at 4 hpf (blastula). We saw a 2-fold increase in *axial* transcripts in the wild-type and the irradiated PKA-ACK expressing embryos, while embryos kept in the dark had normal levels of transcript (Figure 3D). When lysates were collected at 10 hpf, the impact of PKA-ACK decaging on *axial* transcript levels disappears (Figure S10). Unlike the injection of mRNA that continuously produces active caPKA, irradiation of the PKA-ACK acutely generates a fixed amount of active protein that will degrade over time. Most of the leftover ACK will have decaged making it unavailable for incorporation, and any ACK that is still present will only produce caged caPKA. In contrast, the PKA mRNA injection showed a 2-fold reduction in *axial* transcript levels, as was seen in previous reports at this developmental stage.^39,43^ The isolated spike in PKA activity with irradiation of PKA-ACK is a unique property that can offer insight into the role of signaling in specific developmental processes.

**Figure 4:**
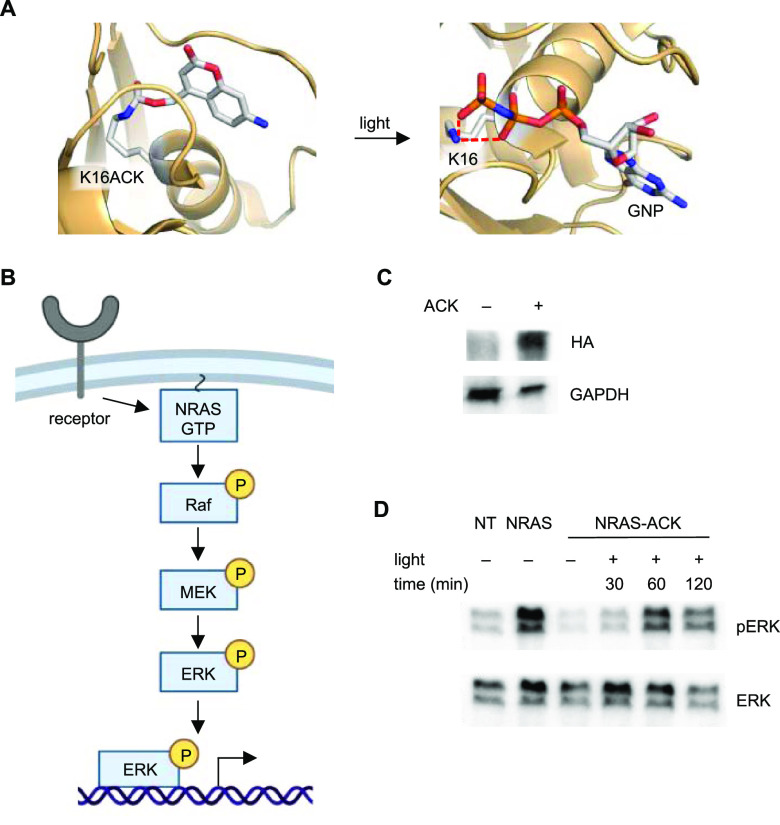
Optical control of a RASopathy mutant of NRAS. (A) Structural representation of the caged NRAS nucleotide-binding site before and after light exposure (models generated from PDB: 5UHV). (B) Diagram of the general RAS/MAPK signaling pathway. (C) Western blot of ACK incorporation into HA-NRAS G60E K16TAG (NRAS-ACK). (D) Western blot of phospho-ERK at different timepoints after irradiation of NRAS-ACK. NT = nontreated embryos.

RAS GTPases are critical upstream players in the RAS/MAPK signaling pathway that are involved in many cell processes from embryo development to cancer.^44,45^ These enzymes activate signaling pathways via interactions with downstream effectors when bound to GTP. RAS enzymes have intrinsic GTPase activity; however, it is slow and is usually assisted by a GTPase activating protein (GAP). Hydrolysis of GTP to GDP induces a conformational change and inactivates its binding to downstream signaling nodes. A guanine nucleotide exchange factor (GEF) then removes the GDP, allowing GTP to freely diffuse back into the active site. Zebrafish have been used to study the impact of certain mutations linked to RASopathies in humans,^46,47^ and one particular mutation is NRAS G60E, which has been shown to cause an increase in RAS/MAPK signaling in cells and zebrafish embryos by reducing binding affinity of GAP.^47,48^ Because RASopathy mutant enzymes cause a wide variety of defects that occur in different tissues and at different timepoints of embryo development, we reasoned that establishing optical control of a RASopathy mutant’s activity could help in studying individual processes with spatiotemporal control without confounding variables that would complicate observations. Structural analysis of NRAS revealed that the conserved critical lysine K16 forms hydrogen bonds with two oxygens from the β- and γ-phosphate of GTP,^49^ important for binding and orienting it for catalysis. We predicted that replacement with ACK would block nucleotide binding due to the large steric bulk of the caging group and disruption of hydrogen bonds (Figure 4A). The constitutive activity imparted by the G60E mutation decouples NRAS and further downstream activation from upstream receptor activation (Figure 4B). We injected mRNA for the NRAS G60E K16UAG mutant along with HCKRS mRNA, PylT, and ACK into embryos, irradiated with 405 nm light at 3 hpf, and collected embryo lysates at 30, 60, and 120 min after irradiation for western blot (Figure 4C,D).

**Figure 5:**
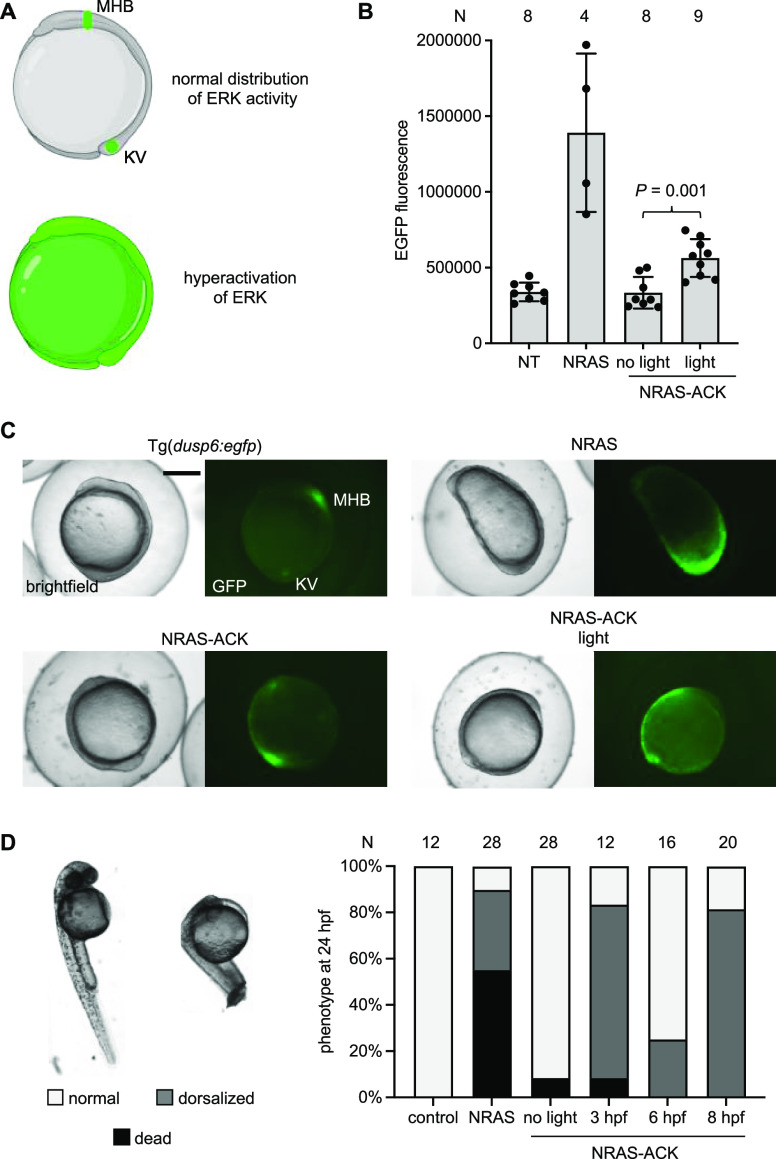
Photoactivation of a RASopathy mutant NRAS. (A) Expected distributions of EGFP in tg(dusp6:egfp) at bud stage (10 hpf). EGFP expression is indicative of ERK activity. At 10 hpf, expression is restricted to Kupffer’s vesicle (KV) and the mid-hindbrain boundary (MHB). (B) EGFP fluorescence was quantified from embryos at 10 hpf for each condition. Irradiation was performed at 6 hpf. Bars represent mean, and error bars represent standard deviation. An unpaired two-tailed Student’s *t*-test was performed between the two samples indicated. NT = nontreated embryos. (C) Representative images of Tg(dusp6:egfp) embryos for each condition. Scale bar = 0.5 mm. (D) Embryos were irradiated at the specified timepoint and scored for dorsalization defects at 24 hpf. Representative images are shown on the left. *N* = number of embryos.

First, we saw incorporation of ACK into NRAS confirming the expression of the caged protein in zebrafish embryos. Immunoblotting for phospho-ERK1/2 (MAPK phosphorylation at T202, Y204/T185, and Y187) showed increased pathway activity by 60 minutes after irradiation, matching NRAS G60E mRNA injection (hereinafter referred to as NRAS), confirming that the RASopathy mutant increased RAS/MAPK signaling and that site-specific incorporation of ACK successfully blocked activity until irradiation. To further validate optical control over this RASopathy mutant enzyme, we used a fluorescent RAS/MAPK signaling reporter zebrafish line, Tg(dusp6:egfp),^50^ to image RAS/MAPK signaling in live embryos. At 10 hpf, RAS/MAPK signaling is restricted to the mid-hindbrain boundary and Kupffer’s vesicle (Figure 5A). Hyperactivation of NRAS activity is expected to increase EGFP fluorescence and disrupt normal patterning of RAS/MAPK signaling. Embryos expressing caged NRAS-ACK were irradiated at 6 hpf and then imaged at 10 hpf. There was significantly increased EGFP fluorescence (Figure 5B), and EGFP fluorescence was present in most of the embryos (Figures 5C and S11). In contrast, embryos expressing NRAS G60E showed severe dorsalization with very high EGFP fluorescence. This dorsalization phenotype was absent in NRAS-ACK embryos irradiated at 6 hpf, as gastrulation has already begun. In support of this, the majority of NRAS-ACK expressing embryos irradiated at 6 or 8 hpf developed normally, while embryos irradiated at 3 hpf displayed gastrulation defects mimicking NRAS G60E expressing embryos by 24 hpf (Figures 5D and S12). Of note, injection of ACK alone and irradiation with 405 nm light at 3 hpf did not elicit any embryo toxicity or deformity (Figures 5D and S12). This led us to speculate that the NRAS RASopathy mutant must elicit its dorsalizing effects prior to gastrulation (around 5 hpf).

**Figure 6:**
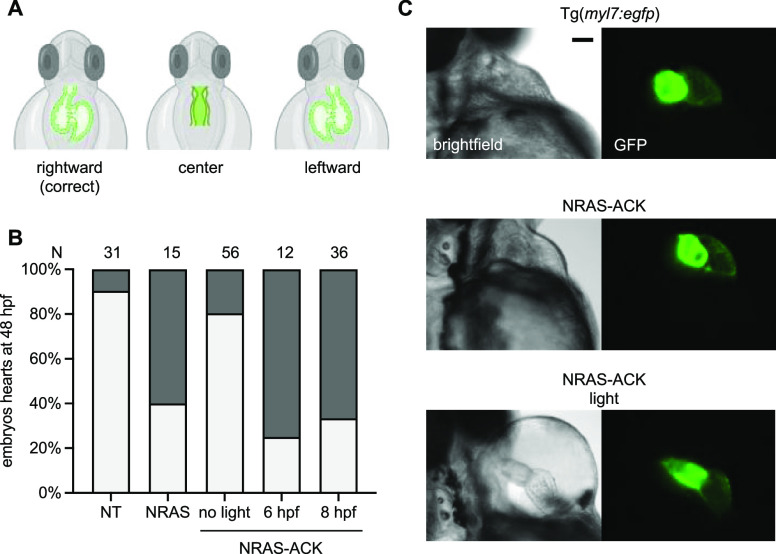
Optical control of RASopathy-induced heart defects. (A) Illustrations of examples of improper heart looping seen in zebrafish embryos. (B) Phenotypic analysis of embryo hearts at 48 hpf. *N* = number of embryos. NT = nontreated embryos. (C) Representative images of Tg(myl7:egfp) embryo hearts at 48 hpf. Pericardial edema is seen in the light-exposed embryos, suggesting impaired cardiac function. Scale bar = 200 μm.

Heart defects are some of the most serious and life-threatening complications in RASopathy patients.^51,52^ These defects can range from septal abnormalities to severe hypertrophic cardiomyopathy. Zebrafish have a two-chambered linear heart tube at 22 hpf that loops by 48 hpf (Figure 6A). We wanted to assess zebrafish embryos for heart defects after expression of the NRAS RASopathy mutant. Therefore, we expressed NRAS-ACK in a myocardial fluorescent transgenic line, Tg(myl7:egfp), to assess if activation of mutant NRAS can cause heart defects. Embryos were irradiated at 6 and 8 hpf followed by imaging of hearts at 48 hpf. We observed an increase in heart defects in embryos expressing the noncaged NRAS (which required injecting a decreased dose of mRNA to avoid embryo death and severe defects) and in embryos expressing the caged NRAS after irradiation, most of which were failures of proper heart looping (Figure 6B,C). A similar incidence of looping defects was seen in zebrafish embryos when expressing other RASopathy mutant GTPases like KRAS and RIT1.^53,54^ These looping defects in the presence of RAS/MAPK activating RASopathy mutants were traced back to impaired cilia function in Kupffer’s Vesicle (KV).^55^ The KV is formed by the dorsal forerunner cells, which are specified in the blastula period of development (between 2 and 5 hpf).^56^ Interestingly, looping defects are seen even with activation of caged NRAS at 8 hpf, suggesting the defect is not due to disruption of specification, but rather due to later processes in KV function, although this would require further investigation. Looping defects are normally not attributed to human patients with RASopathy, rather, they are an inciting factor for the development of most heart defects.^57^ In fact, many complex structural heart defects are linked to disordered looping of the heart, and this is because heart looping is an early and crucial step for establishing the spatial context for subsequent developmental steps.^58^

## Conclusions

Many proteins that bind nucleotides, such as ATP and GTP, rely on a critical lysine residue in order to orient the nucleotide for proper catalysis. This enables the rational design of optically controlled variants of these proteins through genetic code expansion with light-activated lysines. Here, we genetically encoded a new photocaged lysine, ACK (and fluorescent homolog AC_2_K), which shows highly efficient and robust activation through exposure to 405 nm light, and utilized it in the optical control of two enzymes that require nucleotide triphosphate cofactors. One of the biggest advantages of the aminocoumarin moiety is that its absorption of 405 nm light is consistent at a pH of 3–8, compared to the hydroxycoumarin moiety where absorption decreases sharply at a pH below 6.5.^59^ This means that ACK would be expected to consistently decage with the same irradiation stimulus regardless of the extracellular compartment the protein resides in, or the local pH within the protein pocket. Optical control was demonstrated with caPKA, resulting in the induction of gastrulation defect as well as downstream transcriptional changes. Disruption of *axial* is linked to the body axis deformities we observed by 24 hpf.^60^ Interestingly, the transcript quantification results obtained from our PKA photoactivation experiments demonstrate that when PKA is activated at 4 hpf, *axial* transcripts are elevated at 6 hpf and then return to baseline levels by 10 hpf. This acute change in *axial* levels is made possible by the pulse of PKA activity that is sent through the developing embryo, as PKA is rapidly triggered through irradiation, and then activity tapers off as the decaged PKA is degraded. Because the perturbation of *axial* levels during the pre-somitogenic stages (<10 hpf) was enough to phenocopy the constitutively active PKA mRNA injection, we can conclude that the body axis defect is likely limited to disruption during gastrulation (between 6 and 10 hpf). We then applied our caging strategy to the GTPase RAS, which switches between different conformations through hydrolysis of GTP in order to regulate signaling cascades. NRAS G60E is a mildly activated mutant that causes RASopathy in humans and fish. Optical control of this mutant resulted in the expected increase in RAS/MAPK signaling, as well as gastrulation and cardiac defects. Time-resolved activation experiments showed that these gastrulation defects are more common when the enzyme is activated even before gastrulation starts, but not during. This allowed us to decouple the analysis of heart defects from gastrulation defects by activating the NRAS mutant at later stages of development. Here, using temporal control provided by optical stimulation of NRAS, we saw improper heart looping in most embryos that were activated at 6 or 8 hpf. A similar phenotype was seen in other RASopathy mutant-expressing zebrafish embryos and was linked to KV dysfunction.^53,54^ The later timing of NRAS activation and incitement of looping defects that we observed suggest that the KV disruption stems from a process occurring after the formation of the KV progenitor cells, the dorsal forerunner cells. In summary, we demonstrated precise optical control of both kinase and GTPase functions and utilized the temporal control afforded by this methodology to investigate developmental programs. Optical control of NRAS has advantages over small-molecule-induced decaging.^61^ Most important is the fast kinetics of protein activation, which for ACK resulted in full enzymatic function within 2 min of irradiation, while small-molecule-induced activation took about 180 min for full activation in zebrafish embryos as we reported previously (luciferase reporter).^61^ Incorporation of ACK into NRAS provided higher temporal resolution, allowing us to differentiate its role during gastrulation, namely, that NRAS hyperactivity seems to disrupt the early stages of gastrulation most. Furthermore, we expect that our approach of rationally designing photocaged kinases and GTPases through site-specific incorporation of the new caged lysine ACK can be readily applied to most enzymes in both families, making it a useful method for probing the spatiotemporal implications of these proteins in developmental processes in zebrafish.

## Methods

### Zebrafish Care and Microinjection

The zebrafish experiments were performed according to a protocol approved by the Institutional Animal Care and Use Committee (IACUC) at the University of Pittsburgh. Embryos were collected after natural mating. Injection solutions were prepared on ice and a volume of 2 nL was injected into the yolk of 1–2 cell stage embryos using a World Precision Instruments Pneumatic PicoPump injector under a red filter (Pangda, 4331997009) to prevent premature decaging of the UAA. Embryos were incubated in E3 water at 28.5 °C in the dark. For all ACK incorporation experiments, a total of 400 pg of HCKRS mRNA, 16 ng of PylT, and 5 pmol of ACK (from a 100 mM stock in DMSO) were injected along with the mRNA for the protein of interest (injection solution in a total volume of 2–4 μL in Milli-Q water: 200 ng/μL HCKRS mRNA, 200 ng/μL protein of interest mRNA, 8 μg/μL of PylT, and 2.5 mM ACK). For luciferase assays, 400 pg of the fLuc529TAG-rLuc mRNA was injected. For the Cre recombinase experiments, 400 pg of Cre recombinase 201TAG mRNA was injected or 25 pg of wild-type Cre recombinase mRNA for the wild-type control. For caPKA experiments, 400 pg of caPKA 72TAG was injected, or 400 pg of caPKA. For NRAS experiments, 100 pg of NRAS G60E 16TAG mRNA was injected, or 50 pg of NRAS G60E mRNA for the gastrulation experiments, or 25 pg for the heart defect experiments as a positive control. All injection solutions included phenol red at a final concentration of 0.05% as a tracer. As a general approach to how much mRNA was injected, along with the 400 pg of HCKRS mRNA and 16 ng PylT, 400 pg was the maximum amount of the protein of interest mRNA that we injected (more could induce general toxicity in embryos). If this was toxic, we tried titrating the amount of mRNA down to 200, 100, 50, or 25 pg.

### Zebrafish Irradiation and Imaging

For embryo irradiation, a 405 nm LED (Luxeonstar, Luxeon Z, 675 mW) was placed 3 cm above the 35 mm Petri dish containing the embryos suspended in E3 water. Light output at the specimen was measured at 350 mW with a Thorlabs Power Sensor (S170C) and Touch Screen Power and Energy Meter Console (PM200). Stereoscope imaging of embryos was performed with a Leica M205 FA microscope with a DsRed filter (ex: 510–560, em: 590–650), an EGFP filter (ex: 450–490 nm, em: 500–550 nm), and the bright-field channel.

### Luciferase Assays

At 24 hpf, 4 embryos were collected in a 1.5 mL microcentrifuge tube. The water was removed and 1× Passive Lysis Buffer (50 μL, Promega) was added. Embryos were manually homogenized with a p200 pipette tip. Samples were centrifuged at 13 200 rpm for 8 min at 4 °C. A portion of the lysate (30 μL) was added to a white-bottom 96-well plate and loaded into a plate reader with luminometer and autoinjection functions (Tecan Infinite M1000 Pro, injections: 200 μL/s). The Dual-Luciferase Reporter 1000 Assay System (Promega) was used for the assay. An assay program (created using Tecan iControl software) was used to inject fLuc assay reagent (20 μL), pause for 2 s, take a reading, then inject the Stop and Glo rLuc assay reagent (20 μL), pause for 2 s, and take a reading. Autoattenuation mode was on. Corrected fLuc values were calculated by dividing the fLuc value by their rLuc value (fLuc/rLuc). Error bars represent the standard deviation from three calculated fLuc/rLuc ratios from three independent pooled embryo samples (4 embryos/sample).

### Zebrafish Embryo RT-qPCR

We adapted an existing sample preparation protocol for our experiments.^62^ A total of 30–50 shield (6 hpf)- or bud (10 hpf)-stage embryos were collected in a 1.5 mL microcentrifuge tube. The remaining water was removed, and QIAzol Lysis Reagent (250 μL, Qiagen) was added. The embryos were homogenized with a handheld microcentrifuge tube pestle and additional QIAzol lysis reagent (750 μL) was added. The samples were incubated at room temperature for 5 min before adding chloroform (200 μL), mixing, and incubating for an additional 2 min. The samples were centrifuged at 13 200 rpm for 15 min at 4 °C, and the top aqueous layer (600 μL) was transferred to a new tube. TURBO DNase (6 μL, Invitrogen) was added along with 60 μL of 10× TURBO DNase buffer, and the samples were incubated at 37 °C for 15 min to remove any genomic DNA. Ethanol (1 mL, 100%) was added before being transferred to an miRNeasy column (Qiagen) for purification of RNA transcripts following the manufacturer’s protocol. The RNA was eluted into Milli-Q water (20 μL). cDNA was synthesized with the iScript cDNA synthesis kit (Bio-Rad) using 1 μg of RNA template following the manufacturer’s protocol. The cDNA was diluted 1:10 with water, and 2 μL was used in 20 μL qPCR reactions using iTaq Universal SYBR Green Supermix (Bio-Rad) along with primers (Table S1) to amplify zebrafish *axial* (primers 15 and 16) that were reported previously.^63^ As an internal reference control, *ef1a* amplification was used (primers 17 and 18).^64^ The following cycle parameters were used: 95 °C for 30 s, 40 cycles of 95 °C for 5 s, then 56.6 °C for 60 s. The data were analyzed by using the ΔΔCt method.^65^

### Western Blot from Zebrafish Embryo Lysate

At 6 hpf, 100 embryos for each condition were dechorionated and collected in an Eppendorf tube. Next, 200 μL of deyolking buffer (55 mM NaCl, 1.8 mM KCl, 1.25 mM NaHCO_3_) supplemented with 1× protease inhibitor (Roche cOmplete, Mini Protease Inhibitor Cocktail) was added, and yolks were removed by pipetting up and down with a p200 tip about 15 times. The solution was centrifuged at 1100 rcf for 10 min, the supernatant was removed, and the embryo pellet was resuspended and manually homogenized with a pipette tip in T-PER tissue protein extraction reagent (50 μL, Thermo) supplemented with protease inhibitor (Roche cOmplete, Mini Protease Inhibitor Cocktail). Lysates were centrifuged at 16 200 rcf for 5 min at 4 °C, and lysate (30 μL) was mixed with SDS loading buffer (10 μL of 4× loading buffer), denatured at 95 °C for 5 min, and loaded onto a 10% SDS polyacrylamide gel for PAGE (150 V for 90 min). Protein was transferred to a PVDF membrane (80 V for 90 min), blocked with 5% BSA in TBS-T (2 mL) for 1 h at room temperature and incubated with either anti-HA rabbit monoclonal antibody (1:1000, CST #3724), anti-pERK polyclonal rabbit antibody (1:1000, CST #9101), anti-ERK monoclonal mouse antibody (1:1000, SCBT, sc-514302), or anti-GAPDH rabbit polyclonal antibody (1:1000, Proteintech 50-172-6351) in TBS-T containing 5% BSA (2 mL) overnight at 4 °C. Blots were washed with TBS-T (2 mL, 3 × 5 min) and incubated with either anti-rabbit monoclonal antibody-HRP-linked (1:1000 CST #7074) or anti-mouse monoclonal antibody-HRP-linked (1:1000, CST, #7076) in TBS-T (2 mL) at room temperature for 1 h. Blots were washed with TBS-T (2 mL, 3 × 5 min) and then developed with SuperSignal West Pico Chemiluminescent Substrate (2 mL, Thermo) according to the manufacturer’s protocol for 5 min before chemiluminescent imaging on a ChemiDoc imaging system (Bio-Rad).

## Supporting Information Available

Additional methods and figures including Cre recombinase experiments, LC-MS decaging assays, and additional zebrafish images (PDF)
