# Supplementary material for: Genetically Encoded Aminocoumarin Lysine for Optical Control of Protein–Nucleotide Interactions in Zebrafish Embryos
Source: ACS Chem Biol. 2023 Jun 5;18(6):1305–14. doi: 10.1021/acschembio.3c00028 (PMC10278064; doi:10.1021/acschembio.3c00028)
Supplement: Supplementary file 1 — cb3c00028_si_001.pdf [file cb3c00028_si_001.pdf]

Supporting Information

Genetically Encoded Aminocoumarin Lysine for Optical Control of Protein-Nucleotide Interactions in Zebrafish Embryos

Wes Brown[a], Joshua Wesalo[a], Subhas Samanta[a], Ji Luo[a], Steven E. Caldwell[a], Michael Tsang[b], and Alexander Deiters*[a]

[a] Department of Chemistry, University of Pittsburgh, Pittsburgh, PA 15260, United States

[b] Department of Developmental Biology, University of Pittsburgh, Pittsburgh, PA 15260, United States

Contact information: deiters@pitt.edu

Methods

Plasmid construction

The fLuc529TAG-rLuc reporter was cloned into pCS2 using Gibson assembly.[1] To prepare the backbone fragment, pCS2 was digested with BamH1 and EcoR1. CutSmart buffer (1 µL of 10x solution, NEB), followed by BamH1 (0.5 µL, NEB) and EcoR1 (0.5 µL, NEB) were added to a solution of pCS2 (4 µg) in MilliQ water (8 µL). The reaction mixture was incubated for 1 hour at 37 °C. It was then loaded onto a 0.8% agarose-TBE gel and electrophoresed for 40 minutes at 80 volts in TBE buffer supplemented with ethidium bromide (0.2 µg/mL). The linearized vector (4,086 bp) was visualized using a UV transilluminator (365 nm, UVP TFML-20) and excised followed by gel extraction using the GeneJET Gel Extraction kit (Thermo) according to the manufacturer's protocol. The fLuc gene was amplified with primers 1 and 2 from the template pGL3-fLuc (Promega), and the rLuc gene was amplified with primers 3 and 4 from the template pCS2-rLuc WT using PCR. The amplification was performed in a 50 µL reaction containing MilliQ water (34 µL), 10x Taq buffer (KCl, 5 µL), $MgCl_2$ (25 mM, 3 µL), each primer (100 µM, 2.5 µL each), dNTP mix (10 mM, 1 µL), DNA template (10 ng in 1 µL of MilliQ water), and lastly Taq polymerase (1 µL of 5 U/µL solution, Thermo Scientific EP0402). The following thermocycler settings were used: 95 °C for 3 min, 34 cycles of 95 °C for 30 sec, 55 °C for 30 sec, 72 °C for 60 sec, then 72 °C for 5 min. The PCR products were purified by gel electrophoresis using a TBE-agarose gel (0.8%) followed by and gel extraction of the 1,716 bp band for fLuc and 1,002 bp band for rLuc using the GeneJET Gel Extraction kit (Thermo) according to the manufacturer's protocol.

For the Gibson assembly, 5x isothermal reaction buffer was prepared: 1 M Tris–HCl (pH 7.5, 3 mL), 1 M $MgCl_2$ (300 µL), 100 mM dNTP solution (60 µL (contains dATP, dCTP, dGTP, and dTTP), 1 M DTT (300 µL), PEG-8000 (1.5 g), 100 mM NAD^+ (300 µL), and MilliQ water (2,040 µL). The buffer solution was aliquoted and stored at –20 °C. Then, a Gibson assembly master mix was made by mixing isothermal reaction buffer (5x, 40 µL) with T5 exonuclease (1 U/µL, 1.6 µL), Phusion High-Fidelity DNA Polymerase (2 U/µL, 2.5 µL), Taq ligase (40 U/µL, 20 µL), and MilliQ water (85.9 µL). The master mix was aliquoted (15 µL aliquots in PCR tubes) and stored at −20 °C. For each Gibson assembly reaction, a DNA mixture (diluted in MilliQ water (5 µL)) was prepared, then mixed with Gibson assembly master mix. This gives a final reaction volume of 20 µL and final concentrations of 100 mM Tris-HCl pH 7.5, 10 mM $MgCl_2$, 0.2 mM of each dNTP, 10 mM DTT, 5% PEG-8000, 1 mM NAD^+, 4 U/µL Taq DNA ligase, 4 U/mL T5 exonuclease, and 25 U/mL Phusion DNA polymerase. In this case, the purified pCS2 digested vector (100 ng) and 1 molar equivalent of each insert fragment (fLuc529TAG (42 ng) and rLuc (25 ng) fragments) were diluted to 5 µL with MilliQ water, then added to the Gibson assembly master mix (15 µL). The reaction mixture was incubated at 50 °C for 1 hour. Top10 chemically competent cells (50 µL) were heat shock transformed (45 seconds at 42 °C, then 2 minutes on ice) with the Gibson assembly reaction (5 µL), rescued with SOC media (200 µL), all 250 µL were plated on ampicillin (100 µg/mL) containing LB agar plates (10 cm), and colonies were selected after overnight incubation at 37 °C. Overnight cultures in LB media (5 mL, Miller formulation) containing ampicillin (100 µg/mL) were miniprepped using the GeneJET Plasmid Miniprep kit (Thermo) following the manufacturer's protocol to yield pCS2-fLuc529TAG-rLuc. Sequence confirmation of the construct was performed by Sanger sequencing using SP6 and T3 primers (Genewiz, standard primers).

The NRAS G60E and caPKA constructs were cloned into pCS2 with restriction digests. CutSmart buffer (1 µL of 10x solution) was added to a solution of pCS2 (4 µg) in MilliQ water (8 µL) and BamH1 and EcoR1 (for caPKA, NEB, 0.5 µL each) or Cla1 and EcoR1 (for NRAS G60E, NEB,

0.5 µL each) were added. Reaction mixtures were incubated for 1 hour at 37 °C. The caPKA gene was amplified using primers 5 and 6 from a gene fragment (Twist Bioscience) and the NRAS G60E gene was amplified with primers 7 and 8 from a gene fragment (Twist Bioscience) using PCR (50 µL reaction) with recombinant Taq polymerase (Thermo Scientific EP0402) following the manufacturer's protocol. The following thermocycler conditions were used: 95 °C for 3 min, 34 cycles of 95 °C for 30 sec, 55 °C for 30 sec, 72 °C for 60 sec, then 72 °C for 5 min. The PCR reactions were purified using the GeneJET PCR Purification kit (Thermo). The caPKA PCR product was digested with BamH1 and EcoR1 (NEB, 1 µL each in 1x CutSmart buffer) or the NRAS G60E gene product was digested with Cla1 and EcoR1 (NEB, 1 µL each in 1x CutSmart buffer) for 1 hour at 37 °C. The product was purified by agarose gel electrophoresis and gel extraction as described above. T4 DNA Ligase (1 µL) and 10x T4 DNA ligase buffer (1 µL, NEB) were added to a solution of the insert gene (37.5 ng) and digested, purified pCS2 (50 ng) in MilliQ water (8 µL), and the reaction mixture was incubated at room temperature for 1 hour. Top10 chemically competent cells were transformed with the T4 DNA ligase reaction mixture (5 µL) in the same manner as detailed above. Plating, colony selection, miniprep, and Sanger sequencing were performed as detailed above to yield pCS2-caPKA and pCS2-NRAS-G60E.

Site-directed mutagenesis was performed to make the fLuc 529TAG mutation using primers 9 and 10, caPKA K72TAG mutation using primers 11 and 12, and NRAS G60E K16TAG mutation using primers 13 and 14 designed based on a previous report. Briefly, PCR was performed with Phusion polymerase (Thermo) following the manufacturer's protocol. The reaction setup was as follows: MilliQ water (30.5 µL), 5x HF buffer (10 µL), DMSO (1.5 µL), 1 µL of each primer (100 µM in MilliQ water), dNTP mix (10 mM, 1 µL), DNA template (10 ng in 1 µL), and Phusion High-Fidelity Polymerase (2 U/µL, 1 µL). The following thermocycler parameters were used: 95 °C for 3 min, 25 cycles of 95 °C for 30 sec, 55 °C for 30 sec, 72 °C for 2.5 min, then 72 °C for 5 min. CutSmart buffer (5.5 µL of 10x solution) followed by DpnI (1 µL, NEB) was added to the 50 µL reaction mixture, and the mixture was incubated for 2 hours at 37 °C to remove template DNA. After incubation, Top10 chemically competent cells (50 µL) were incubated with the resulting digestion product (5 µL) and heat shock transformed and plated as detailed above. Colonies were selected, grown overnight, miniprepped, and sequence verified as detailed above. Plasmid constructs generated: pCS2-fLuc 529TAG-rLuc, pCS2-caPKA, pCS2-caPKA 72TAG, pCS2-NRAS G60E, pCS2-NRAS G60E 16TAG,

Expression and purification of sfGFP-Y151ACK and sfGFP-Y151AC$_2$K in bacteria

Top10 cells transformed with pBK-EV12[2] and sfGFP-Y151TAG-PylT[3] were inoculated from a glycerol stock into LB media (Miller formulation, 2 mL) containing kanamycin (50 µg/mL) and tetracycline (25 µg/mL). After overnight growth (37 °C, 280 rpm), the saturated culture (0.9 mL) was diluted in LB media (45 mL) containing kanamycin (50 µg/mL) and tetracycline (25 µg/mL). The resulting cell suspension was mixed thoroughly by pipetting it up and down five times, and was split in half by pipetting equal portions (20 mL each) into three separate 125 mL Erlenmeyer flasks. The cultures were incubated at 37 °C, 280 rpm until reaching OD_{600} 0.3. At this time, ACK or AC$_2$K (200 µL of a 100 mM stock solution in DMSO for a 1 mM final concentration) were added to two cultures, and DMSO vehicle (200 µL) was added to the other. The flasks were wrapped with aluminum foil to exclude light, and were returned to the incubator for 25 min, after which arabinose (200 µL of 20% (w/v) solution for a 0.2% final concentration) was added to induce protein expression. The cultures were incubated overnight (37 °C, 280 rpm), then were transferred to 50 mL conical tubes and were pelleted by centrifugation (4,500 rcf, 10 min, swinging bucket rotor). The pellets were washed with PBS (20 mL) and were resuspended in 5 mL of ice-cold lysis buffer (50 mM sodium phosphate, pH 8.0) containing Triton X-100 (0.1%) and lysozyme (1

mg/mL). After gentle mixing on ice for 60 min, the cell suspensions were sonicated on ice (6 cycles of 30 s on, 30 s off, 45% amplitude, Fisher Scientific 550 Sonic Dismembrator with micro-tip). The resulting lysates were clarified by centrifugation at 4 °C (13,000 rcf, 10 min). The supernatants were transferred to 15 mL conical tubes and 100 µL of Ni-NTA resin (Qiagen) was added to each sample. The mixture was gently rocked on ice for 2 h. The resin was collected by centrifugation at 4 °C (500 rcf, 10 min), washed with lysis buffer (2 x 400 µL), washed with lysis buffer supplemented with imidazole (20 mM, 2 x 400 µL), and eluted with lysis buffer supplemented with imidazole (250 mM, 400 µL). The purified protein was analyzed by SDS-PAGE (10%) stained with Coomassie Brilliant Blue G (1 mg/mL in 7:2:1 dH_2O:methanol:acetic acid).

Whole-protein MALDI-TOF-MS

Sample preparation. A µC_{18} resin Zip-Tip (Millipore-Sigma Cat. No. ZTC18M) was placed on a P20 micropipette set to 10 µL per the manufacturer's instructions. The following procedures were then carried out without allowing the tip to dry out once it was wetted. The resin was wetted by drawing up HPLC-grade MeCN (10 µL), and expelling 8-9 µL of it onto a Kimwipe, and repeating the process 4-5 times. The resin bed was then equilibrated with 10 washes of 0.1% TFA in Milli-Q water by the same procedure. A sample of sfGFP-Y151ACK (10 µL) in elution buffer (see above) was mixed with 20% TFA in Milli-Q water (2 µL) in a microcentrifuge tube. The sample-TFA mixture was then loaded onto the resin by pipetting it back-and-forth, up through the resin and back into the microcentrifuge tube 10-15 times, taking care to avoid fully drying out the resin each time. The resin was washed with 0.1% TFA in Milli-Q water (10 x 10 µL), each time expelling 8-9 µL of the wash onto a Kimwipe. SA elution solution (3 µL) consisting of 10 mg/mL sinapic acid in 1:1 MeCN:0.1% TFA (aq) was pipetted into another PCR tube. The purified protein sample for MS analysis was eluted by passing through the tip back-and-forth 15 times.

MALDI-TOF-MS analysis. A stainless steel target plate (Bruker MSP 96) was pre-spotted with SA elution solution (0.5 µL). The plate was dried in open air for 10 min, and the resulting crystals were gently wiped with a Kimwipe, leaving some on the target. The eluted sample solution was pipetted portion wise on top of the wiped matrix crystals (3 x 1 µL), allowing the sample to air-dry completely in a dark drawer for 10 min between each addition. Samples were analyzed on a Bruker Daltonics UltrafleXtreme MALDI-TOF-MS in linear positive mode.

Whole-protein ESI-MS

Analysis was performed on a Thermo Scientific Q-Exactive Orbitrap mass spectrometer connected to a Dionex Ultimate 3000 UHPLC system. The sample was analyzed with a ProSwift RP-10R 1 mm x 5 cm column, flow rate 200 µL/min and a 26→80% MeCN in 0.1% HCOOH gradient over 30 min. The mass spectrometer was operated in positive-ion mode with a capillary voltage of 3.5 kV and resolution set to 17,500. Sheath gas, auxiliary gas, and sweep gas flow rates were 35, 10, and 5 L/min, respectively. Source temperature was 250 °C. The S-lens RF level voltage was 50 V and the ion transfer tube temperature was 250 °C. The instrument was tuned and calibrated with Pierce LTQ ESI positive ion calibration solution (Thermo Scientific) and the data were collected for *m*/*z* values ranging from 500-3000. Xcalibur 3.0.63 and Protein Deconvolution 3.0 software were used for data analysis and deconvolution.

Genetic encoding of aminocoumarin lysines in mCherry-TAG-EGFP-HA reporter in HEK293T cells — imaging and western blot.

HEK293T cells (American Type Culture Collection) were seeded at 160,000 per well into a 6-well plate (Greiner Bio-One 657160) that had been coated with poly-D-lysine (HBr salt, MP Biomedicals, 70-150 kDa). Cells were grown for 18 h (37 ºC, 5% CO_2) to ~80% confluency in Dulbecco's Modified Eagle's Medium (DMEM; HyClone Laboratories, GE Life Sciences) supplemented with fetal bovine serum (FBS, 10% (v/v), Sigma-Aldrich), penicillin (100 U/mL, Corning Cellgro), and streptomycin (100 µg/mL, Corning Cellgro). To transfect the cells, a solution of linear polyethylenimine (1 mg/mL, LPEI, Polysciences) was diluted to 0.33 mg/mL with prewarmed Opti-MEM media (Gibco). The resulting solution (10 µL) was added to solutions of mini-prepped (Omega Bio-tek) plasmid DNA (1.5 µg each) in Opti-MEM media (200 µL). The plasmids were pE363-mCherry-TAG-EGFP-HA-$PylT_4$ (provided by the laboratory of Jason Chin) as the reporter, and pE323-(IPYE)HCKRS-$PylT_4$ encoding the PylRS and additional copies of PylT.[4] Cell culture media was replaced with antibiotic-free DMEM (1.8 mL) supplemented with FBS (10% (v/v)) in the presence or absence of the UAA (ACK, AC_2K, or HCK, all 0.25 mM) or DMSO vehicle (0.25% (v/v)). The transfection reagent mixtures were incubated for 15 min before adding to the cells, at which time 180 µL of the reagent mixture was added per well. The reagent mixture was added by dispensing a small droplet out of the tip of the pipette tip and touching it to the surface of the media over the cells. Once the reagent was added to all wells, the plate was gently swirled for ~5 seconds and placed in the incubator (37 ºC, 5% CO_2). After 48 h, cells were washed with warm PBS (2 x 1.25 mL per well) and changed to fresh PBS (2 mL) to check for protein expression by imaging. The fusion protein was visualized by epi-fluorescence microscopy using a Zeiss Axio Observer Z1 (10x objective, Plan-Apochromat 0.4 NA) with a short mercury arc bulb (Bulbman Inc Osram) in a Zeiss HBO 100 W housing and Zen 2 Blue Edition software. Filter cubes were excitation (ex); BP550/25, emission (em), BP605/70 for mCherry; ex, BP470/40; em, BP525/50 for EGFP; and ex, G 365; em, BP 445/50 for HCK, ACK and AC_2K.

Cell lysis. After imaging, cells were cooled on ice, PBS was removed, and cells and lysed in ice-cold mammalian protein extraction buffer (250 µL) (GE Life Sciences) supplemented with Halt Protease Inhibitor Cocktail (Thermo Scientific) on ice with orbital shaking for 20 min. The lysed cells were scraped from the plates using P1000 pipette tips. Lysates were pipetted into microcentrifuge tubes, and were clarified by centrifugation (21,000 rcf, 20 min, 4 ºC).

Western blot. Supernatants (50 µL) were mixed with 6x Laemmli sample buffer (12 µL) and heated to 98 ºC for 10 min. After cooling to room temperature, the samples (28.5 µL of each) were loaded into 1.5 mm 10% polyacrylamide gels alongside PageRuler molecular weight marker (7 µL, Thermo Scientific). Gels were run at 60 V for 10 min, then 80 V for 10 min, then 125 V for 90 min. Proteins were transferred to PVDF membranes (Immobilon-P, EMD Millipore) for 2 h, 45 min at 75 V at 0 ºC. The membrane was blocked with 5% (w/v) powdered nonfat milk in TBS-T for 1 h. Membranes were rinsed with TBS-T (3 x 5 min) and incubated in anti-GAPDH (Santa Cruz Biotechnology, Dallas, TX, sc-365062, mouse mAb, diluted 1:2,000 in 5% (w/v) milk in TBS-T), anti-β-actin (Santa Cruz Biotechnology, Dallas, TX, mouse mAb, diluted in 5% (w/v) milk in TBS-T)) anti-HA (Cell Signaling Technology, Danvers, MA, C29F4, rabbit mAb, diluted 1:1,500 in 5% (w/v) BSA in TBS-T) antibody solutions for 8 h at 4 ºC. Membranes were washed with TBS-T (5 × 5 min) and incubated with secondary antibodies for 1 h at ambient temperature (1. goat anti-mouse IgG-HRP, Santa Cruz Biotechnology, sc-2005, diluted 1:20,000 in TBS-T; 2. goat anti-rabbit IgG-HRP, Santa Cruz Biotechnology, 7074S, diluted 1:5,000 in TBS-T). Membranes were washed with TBS-T (5 × 5 min) and were imaged with West Pico Chemiluminescence Substrate (Thermo Scientific, Waltham, MA) using a ChemiDoc XRS+ system with Image Lab 6.0 software (Bio-Rad, Hercules, CA) in high resolution chemiluminescence mode (2 × 2 binning).

Genetic encoding of AC_2K in various reporters in NIH 3T3 cells.

NIH 3T3 cells (American Type Culture Collection) were plated (40,000/well) in an 8-well chamber slide (Cellvis 8-well chambered cover glass, #1.5, Cat. No. C8-1.5H-N) that had been coated with poly-D-lysine (HBr salt, MP Biomedicals, 70-150 kDa). The cells were incubated (37 ºC, 5% CO_2) in DMEM (HyClone Laboratories, GE Life Sciences) supplemented with fetal bovine serum (FBS, 10% (v/v), Sigma-Aldrich), penicillin (100 U/mL, Corning Cellgro), and streptomycin (100 µg/mL, Corning Cellgro) for 24 h, after which they were ~85% confluent. The cells were transfected with pE323-(IPYE)HCKRS-$PylT_4$ and pNLS-TAG-GFP using Lipofectamine 3000 (Invitrogen). For each well, a total of 400 ng DNA was used (comprising 200 ng of each of the two plasmids). The DNA was diluted in Opti-MEM (10 µL/well). Next, P3000 reagent (Invitrogen, 0.4 µL/well) was added. Lipofectamine 3000 (0.4 µL/well) was diluted in Opti-MEM (10 µL/well), and DNA was added to the diluted Lipofectamine 3000 solution (10 µL) and mixed by pipetting up and down 6-8 times. The mixture was incubated 10-15 min at ambient temperature. Meanwhile, the media for the cells was replaced with DMEM +10% FBS (400 µL) supplemented with **AC_2K** (0.25 mM). Then, the transfection reagent (20 µL/well) was added to the cells dropwise by dispensing small droplets onto the pipette tip and touching each one to the media surface.

After transfection, cells were incubated for 48 h (37 ºC, 5% CO_2), and were incubated in 3 changes of fresh DMEM +10% FBS (3 x 400 µL, 30 min each) to remove excess UAA and reduce background. Media was removed and replaced with prewarmed PBS (400 µL). Cells were imaged using a Zeiss Axio Observer Z1 with 20x objective, numerical aperture 0.8 Plan-Apochromat M27 with a short mercury arc bulb (Bulbman Inc Osram) in a Zeiss HBO 100 W housing using Zen 2 Blue Edition software. Filter cubes were excitation (ex); BP550/25, emission (em), BP605/70 for mCherry; ex, BP470/40; em, BP525/50 for EGFP; and ex, G 365; em, BP 445/50 for HCK, ACK and AC_2K.

Genetic encoding of ACK and AC_2K in HeLa cells.

HeLa cells (American Type Culture Collection) were plated in a 16-well chamber slide (Nunc Lab-Tek, Thermo Scientific 178599) at 20,000/well. The cells were incubated in DMEM +10% FBS +penicillin-streptomycin (400 µL/well) for 18 h, after which they were ~75% confluent. Cells were transfected using FuGENE 4K with pE323-(IPYE)HCKRS-$PylT_4$[4] and pNLS-TAG-EGFP.[2] Media was changed to 130 µL of DMEM +10% FBS supplemented with **ACK** (0.25 mM) or **AC_2K** (0.2 mM) 30 minutes before transfection. To transfect the cells, plasmids (200 ng total per well, with 100 ng of pE323-(IPYE)HCKRS-$PylT_4$ and 100 ng of the pNLS-TAG-EGFP reporter plasmid) were diluted in Opti-MEM (5 µL/well). FuGENE 4K (0.4 µL/well) was then added to the diluted DNA. After 48 h of growth, cells were washed/incubated (3 x 30 min incubations in 200 µL media) with FluoroBrite DMEM supplemented with 2% FBS. The cells were checked for successful protein expression by fluorescence microscopy (before proceeding with fixation and staining.

Fixation and staining. Cells were fixed by removing half of the media (100 µL) and adding 100 µL of 2x paraformaldehyde (8% (w/v)) solution in PBS, then incubating at 37 °C for 20 min. Cells were washed with PBS (3 x 200 µL) and permeabilized with 0.1% Triton X-100 in PBS (200 µL, 15 min, room temperature) before staining. Cells were blocked with 1% BSA in PBS for 40 min (200 µL), then were stained with rhodamine-phalloidin (Invitrogen, Cat. No. R415). The rhodamine-phalloidin conjugate that had been reconstituted in MeOH (1.5 mL, for 200 U/mL) according to the manufacturer’s instructions. This stock solution (9.2 µL) was diluted 45-fold with PBS (400 µL) before use, and was added to each well to be stained (125 µL/well). Cells were incubated in this solution for 30 min. Stained cells were washed with PBS (3 x 200 µL).

Mounting. Once the staining was complete, all PBS was removed. The chamber dividers were snapped off, and the rubber gasket beneath it was carefully removed using a razor blade to free the edge and gentle traction with forceps. The slide was air-dried for 2 h in a drawer to exclude light and dust. A small drop of Gelvatol reagent (Center for Biologic Imaging, University of Pittsburgh; protocol for preparation below) was applied to each circle of cells using a 3 mL syringe. A 22 x 60 mm #1.5 coverslip was gently applied starting at a ~15° angle, taking care to push all air bubbles out to the far edge while lowering the slide to a horizontal position. The mounted slide was set at 4 °C overnight (laying flat). The following day, the edges were sealed by applying a minimal quantity of Entellan ("New" formula, Millipore-Sigma) with a cotton swab to the edges, curing for 5 h at room temperature in the absence of light (in a box), and storing at 4 °C in the exclusion of light thereafter. The slide was equilibrated to room temperature for 30-60 min before imaging to avoid condensation on the surface. Any dust was gently wiped from the slide with a lens tissue, taking care not to put pressure on the samples.

The protocol for Gelvatol preparation is available on the CBI website (https://cbi-pitt.webflow.io/protocols). To prepare gelvatol, mix water (104 mL), tris (0.2 M, pH 8.5, 212 mL), and glycerol (84 mL) on a hot plate with stirring at 75 °C. Polyvinyl alcohol (Sigma P-8136 must be used; 42.0 g) is added in portions of ca. 5 g each, waiting until complete dissolution between additions (approximately 1 h per addition). The resulting mixture is clarified by centrifugation (5,000 rcf, 15 min), aliquoted, and stored at 4 °C. Aliquouts were stored in 3 or 5 mL syringes with caps or with the orifice plugged with the narrow end of a P2 pipette tip; Gelvatol may be dispensed directly from these syringes onto slides.

Imaging. Cells were imaged with a Zeiss Axio Observer Z1 with an Andor Zyla 4.2 camera, Excelitas X-Cite 120 LED Boost light source, and Slidebook 6 software (3i). Two objectives were used: 40x air (NA 0.6 LD Plan-Neofluar) and 63x (oil immersion, NA 1.4 Plan-Apochromat. Zeiss Immersol 518F immersion oil for fluorescence microscopy (Zeiss Cat. No. 1262466A, refractive index 1.518) was used with the 63x lens. Filter cubes were used for fluorescence microscopy for GFP (Chroma filter 49002, Ex. ET 470/40; Em. ET 525/50), for mCherry (Zeiss filter set 43, Ex. BP 550/25; Em. 605/70), and for ACK and AC_2K (Chroma filter 49028, Ex. ET 395/25, Em. ET 460/50). Image deconvolution was performed in Slidebook 6 (3i) with a PSF estimated based on the NA, index of refraction, and working distance of the objective lens when required by the algorithm. For ACK, No Neighbors Deconvolution was performed with a subtraction constant of 0.9, theoretical spacing of 0.5, and edge padding of x: 78, y: 56 (default values). For AC_2K, the Autoquant Blind Widefield Deconvolution algorithm was used with the maximum number of iterations set to 10; edge padding and correction for spherical aberration were disabled.

***In vitro* transcription**

To synthesize mRNA, 10x CutSmart buffer (1 μL) and Not1-HF (1 μL, 20 U/μL, NEB) was added to a solution of the corresponding pCS2 plasmid (4 μg) in MilliQ water (8 μL). The reaction mixture was incubated at 37 °C for 1 hour. The reaction mixture was diluted with water (40 μL) to a total volume of 50 μL, and the product was purified by phenol:chloroform:isoamyl alcohol (PCIA) extraction. Briefly, PCIA (50 μL) was added to the reaction mixture and the suspension was vortexed and then centrifuged (16,200 rcf for 1 minute). The top aqueous layer was carefully pipetted off into a new microcentrifuge tube. Sodium acetate (3 M, 1/10 volume of DNA solution, 5 μL) was then added, and the DNA was precipitated with 100% ethanol (2.5x volume of the DNA and sodium acetate solution, 138 μL) at –20 °C overnight, then pelleted at 16,200 rcf for 5 minutes. The DNA pellet was washed with 70% ethanol, pelleted by repeating the centrifugation step, and the supernatant was removed by pipette. The pellet was dried at room temp with the cap open for 5 minutes before dissolving it in MilliQ water (20 μL).

The linearized plasmid (1 µg) was used in a 20 µL SP6 mMessage mMachine *in vitro* transcription reaction (Thermo) following the manufacturer's instructions. The reaction mixture contained linearized DNA template (1 µg), NTP/cap mix (10 µL), 10x buffer (2 µL), and SP6 RNA polymerase (2 µL) was diluted up to a final volume of 20 µL with MilliQ water. Reaction mixtures were incubated at 37 °C for 1-2 hours. Reaction mixtures were treated with Turbo DNase (Thermo, 1 µL), purified by PCIA extraction, ethanol precipitated, and dissolved in MilliQ water (20 µL) as described above and stored at –80 °C. A 1:10 dilution of the RNA was made in MilliQ water for concentration measurement with a Nanodrop and quality assessment by TBE-agarose gel electrophoresis (0.8%, 80 V, 45 min) and ethidium bromide staining (0.2 µg/mL included in TBE-agarose gel, **Figure S8**).

LC-MS decaging assay (pH-dependent)

Sodium phosphate buffer solutions (100 mM) (pH 5.0, 5.5, 6.0, 6.5, 7.0, 7.5, and 8.0) were prepared, and HCK or ACK was diluted to 100 µM in the buffer from a 100 mM DMSO stock. For each pH, duplicate solutions (50 µL each) were transferred into polypropylene autosampler vials (Chemglass, Cat. No. CV-1007-1232). The 405 nm LED (Luxeonstar, Luxeon Z, 675 mW) was held about 3 cm above the solution (350 mW, measured with Thorlabs Power Sensor (S170C) and Touch Screen Power and Energy Meter Console (PM200)), and samples were irradiated for 10 seconds. Samples were diluted to a common matrix with the addition of 10x PBS, pH 7.4 (10 µL) before analysis. Photolysis products were analyzed by LC-MS using a Shimadzu 2020 instrument. A 2.1 mm x 100 mm Hypersil C_{18} GOLD column (1.9 µm) at 40 °C was used. The injection volume was set to 3 µL, and a 3→45% MeCN in 0.1% HCOOH gradient was run over 10 minutes. EICs were obtained for the UAAs and for the released coumarin products in positive mode. The masses were $[M+H]^+$ = 365 for HCK and $[M+H]^+$ = 193 for its product, and $[M+H]^+$ = 364 and $[M+H]^+$ = 192 for ACK. The retention times were 6.01 min for HCK and 5.96 min for the released product, and 5.85 min for ACK and 5.58 min for the released product. Peak height for was calculated automatically in LabSolutions software (Shimadzu) using File > Print Graph Image > PDF Output.

LC-MS comparison assay (pH 7.4)

Methylation. Since the lysine methyl ester provided a much stronger signal in the LC-MS than the free amino acid, aliquots of ACK and HCK (20 µL, 2.0 nmol) were added to dry MeOH (200 µL) in flame-dried glass vials. The solutions were cooled to 0 °C and trimethylsilyl-diazomethane (20 µL, 40.0 nmol, 2 M in Et_2O) was added slowly. The vials were wrapped in aluminum foil and allowed to stir at room temperature overnight. Upon completion the volatiles were evaporated and the resulting DMSO solutions were used directly in the assay.

LC-MS analysis. HCK-OMe and ACK-OMe were diluted to 100 µM in PBS (pH 7.4, containing 2% DMSO) from the 100 mM methylated stock solutions. An internal standard of *N*-Boc-Lys-OMe was added at 100 µM to each sample. Duplicate solutions (75 µL each) were transferred into polypropylene autosampler vials (Chemglass, Cat. No. CV-1007-1232) and a 405 nm or 365 nm LED was held about 3 cm above the solution (350 mW, measured with Thorlabs Power Sensor (S170C) and Touch Screen Power and Energy Meter Console (PM200)) for the indicated time. Samples were diluted with addition of 10x PBS, pH 7.4 (15 µL) before analysis to recapitulate the matrix from the pH-dependent assay. Photolysis products were analyzed by LC-MS using a Shimadzu 2020 instrument. A 2.1 mm x 100 mm Hypersil C_{18} GOLD column (1.9 µm) at 40 °C was used. The injection volume was set to 10 µL, and a 3% → 45% MeCN containing 0.1%

HCOOH gradient was run over 10 minutes. EICs were obtained for the methylated ACK and HCK and for the released Lys-OMe in positive ion mode. The masses were $[M+H]^+$ = 379 for HCK-OMe, $[M+H]^+$ = 378 for ACK-OMe, and $[M+H]^+$ = 161 for Lys-OMe. The retention times were 6.97 min for HCK-OMe, 6.83 min for ACK-OMe, and 1.52 min for Lys-OMe. Peak height was calculated automatically in LabSolutions software (Shimadzu) using File > Print Graph Image > PDF Output.

Data Analysis. Initial concentrations of HCK-OMe and ACK-OMe were determined by measuring the concentration of HCK and ACK before and after reaction with trimethylsilyl-diazomethane. The difference represents the HCK-OMe and ACK-OMe concentration. This was achieved by acquiring standard curves from stock solutions (0, 25, 50, and 100 μM) that were prepared in the same way as above. Using this method, we found that the concentration of HCK-OMe was about 76 μM and the concentration of ACK-OMe was about 59 μM before irradiation. The concentration of Lys-OMe released from each irradiation time was calculated using a standard curve for commercially available Lys-OMe (0, 25, 50, and 100 μM). Finally, the percentage of released Lys-OMe for each reaction was obtained by dividing the concentration of Lys-OMe by the concentration of HCK-OMe and ACK-OMe before irradiation and multiplied by 100%.

Generating HCKRS docking models

The docking of HCK and ACK into the HCKRS binding pocket were performed using Chimera (UCSF) and Autodock Vina software. First, the PylRS synthetase protein model was opened in Chimera (PDB: 2Q7H) and the appropriate HCKRS pocket mutations were introduced using the “change rotamer” function (Tools>Structure editing>Rotamers, Y271A, L274M). The “Dock prep” function was performed using the default conditions (Tools>Structure editing>Dock prep), then the Autodock Vina tool was opened (Tools>Surface/Binding analysis>Autodock Vina). The PDB model of HCK or ACK was selected as a ligand, and the HCKRS as the receptor, then the docking selection box was adjusted around the UAA binding pocket (as visualized by the pyrrolysyl UAA residing in the pocket from the original PDB model). The pyrrolysyl UAA was then deleted, leaving an open pocket for docking. The docking process was performed, generating several models. Only the model with the correct orientation of the UAA in the pocket was used for comparison between HCK and ACK.

Synthesis of caged 7-aminocoumarin lysine (ACK)

7-Aminocoumarin lysine **1** was synthesized from 3-aminophenol via the six step reaction sequence shown in **Scheme S1.** The first four steps, adapted from the literature,[5] were to construct the intermediate 7-aminocoumarin alcohol **4** from 3-aminophenol. The alcohol **4** was reacted with 1,1'-carbonyldiimidazole, followed by Boc-L-lysine-OH, to yield the coumarin-lysine **3** in 74% yield over two steps. Subsequent Boc group deprotection using TFA in the presence of TES as a cation scavenger led to the 7-aminocoumarin lysine amino acid **1** as a TFA salt in 86% yield.

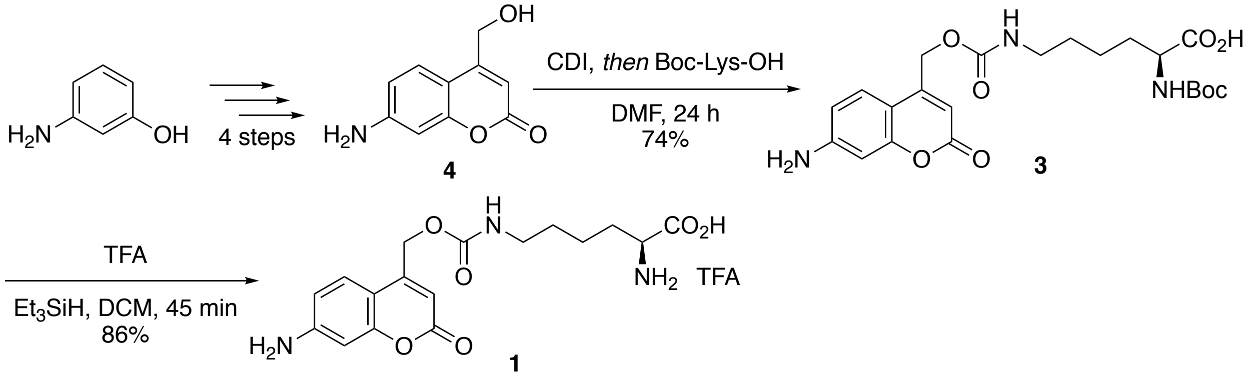

Scheme S1. Synthesis of the 7-aminocoumarin lysine **1**.

***N*[6]-(((7-Amino-2-oxo-2H-chromen-4-yl)methoxy)carbonyl)-*N*[2]-(*tert*-butoxycarbonyl)-L-lysine (3)**: 1,1'-Carbonyldiimidazole (54 mg, 0.34 mmol) was added to a solution of the coumarin alcohol **4** (53 mg**,** 0.28 mmol) in anhydrous DMF (1.0 mL). The reaction mixture was stirred at room temperature for an hour under a nitrogen atmosphere. Then, Boc-L-lysine-OH (84 mg, 0.36 mmol) was added as a solution in anhydrous DMF (0.5 mL). The reaction mixture was stirred at room temperature for 24 h. After completion of the reaction as judged by TLC analysis, the reaction mixture was concentrated under vacuum and the product was purified by flash chromatography on silica gel, eluting with 95:5→90:10 DCM:MeOH to give **3** as a white solid (89 mg, 74%). ^{1}H NMR (500 MHz, CD_3OD): δ 7.28 (d, J = 8.5 Hz, 1H), 6.54 (dd, J = 8.5 Hz, J_2 = 2.0 Hz, 1H), 6.42 (d, J = 2.0 Hz, 1H), 5.96 (s, 1H), 5.16 (s, 2H), 3.93-3.95 (m, 1H), 3.04-3.08 (m, 2H), 1.59-1.78 (m, 1H), 1.51-1.60 (m, 2H), 1.40-1.50 (m, 3H), 1.33 (s, 9H); ^{13}C NMR (125 MHz, CD_3OD): δ 177.4, 164.3, 158.0, 157.9, 156.1, 154.5, 153.0, 125.9, 113.1, 108.1, 105.8, 100.6, 80.3, 62.6, 55.3, 41.6, 32.7, 30.3, 28.7, 24.0.

***N*[6]-(((7-Aamino-2-oxo-2H-chromen-4-yl)methoxy)carbonyl)-L-lysine** (**1**): TFA (0.5 mL) was added to a solution of **3** (88 mg, 0.20 mmol) and triethylsilane (64 µL, 2.2 mmol) in DCM (0.5 mL), and the resulting mixture was stirred at room temperature under a nitrogen atmosphere for 45 min. The reaction mixture was concentrated under vacuum. The residue was dissolved in MeOH (2 mL) and concentrated under reduced pressure. This process was repeated one more time to remove residual TFA. Finally, the crude product was dissolved in a minimal volume of MeOH and precipitated in Et_2O under vigorous stirring. The precipitate was collected, and dissolved in a minimal volume of MeOH followed by precipitation in Et_2O once more. The resulting precipitate was collected, redissolved in a minimal volume of MeOH, and precipitated in Et_2O a third time to furnish coumarin lysine **1** (75 mg, 86%) as an amorphous white solid. ^{1}H NMR (400 MHz, D_2O): δ 7.30 (d, J = 8.4 Hz, 1H), 6.71 (d, J = 8.4 Hz, 1H), 6.61 (s, 1H), 6.03 (s, 1H), 5.11 (s, 2H), 3.78

(t, J = 6.0 Hz, 1H), 3.09 (t, J = 6.4 Hz, 2H), 1.74-1.87 (m, 2H), 1.45-1.52 (m, 2H), 1.34-1.40 (m, 2H); ^{13}C NMR (125 MHz, DMSO-d_6): δ 171.5, 161.2, 156.0, 155.9, 153.7, 152.7, 125.7, 111.8, 106.3, 104.6, 99.1, 61.3, 53.1, 30.5, 29.4, 22.3; HRMS (ESI+) calcd for $C_{17}H_{22}N_3O_6$ $[M+H]^+$ 364.1503, found 364.1499.

Synthesis of fluorescent 7-aminocoumarin lysine (AC$_2$K)

Fluorescent 7-Aminocoumarin lysine **2** was synthesized from 3-bromophenol via the eight step reaction sequence shown in **Scheme S2**. Pechmann condensation of 3-oxopentanedioic acid and 3-bromophenol in the presence of methanesulfonic acid led to 2-(7-bromo-2-oxo-2H-chromen-4-yl)acetic acid **11**. The carboxylic group of **11** was then reduced to alcohol **10** using $BH_3{\cdot}Me_2S$. After TBDMS protection of the alcohol, it was subjected to Buchwald-Hartwig amination leading to a Boc protected 7-aminocoumarin derivative **8**. Next, the TBDMS group was released with the aid of TBAF and the resulting free alcohol **7** was converted to *p*-nitrophenyl carbonate **6** by reaction with 4-nitrophenyl chloroformate. Finally, the Boc-L-lysine-OH was reacted with the carbonate **6** to yield the coumarin-lysine **5,** which upon treatment with 50% TFA led to desired fluorescent 7-aminocoumarin lysine amino acid **2** as a TFA salt in 86% yield.

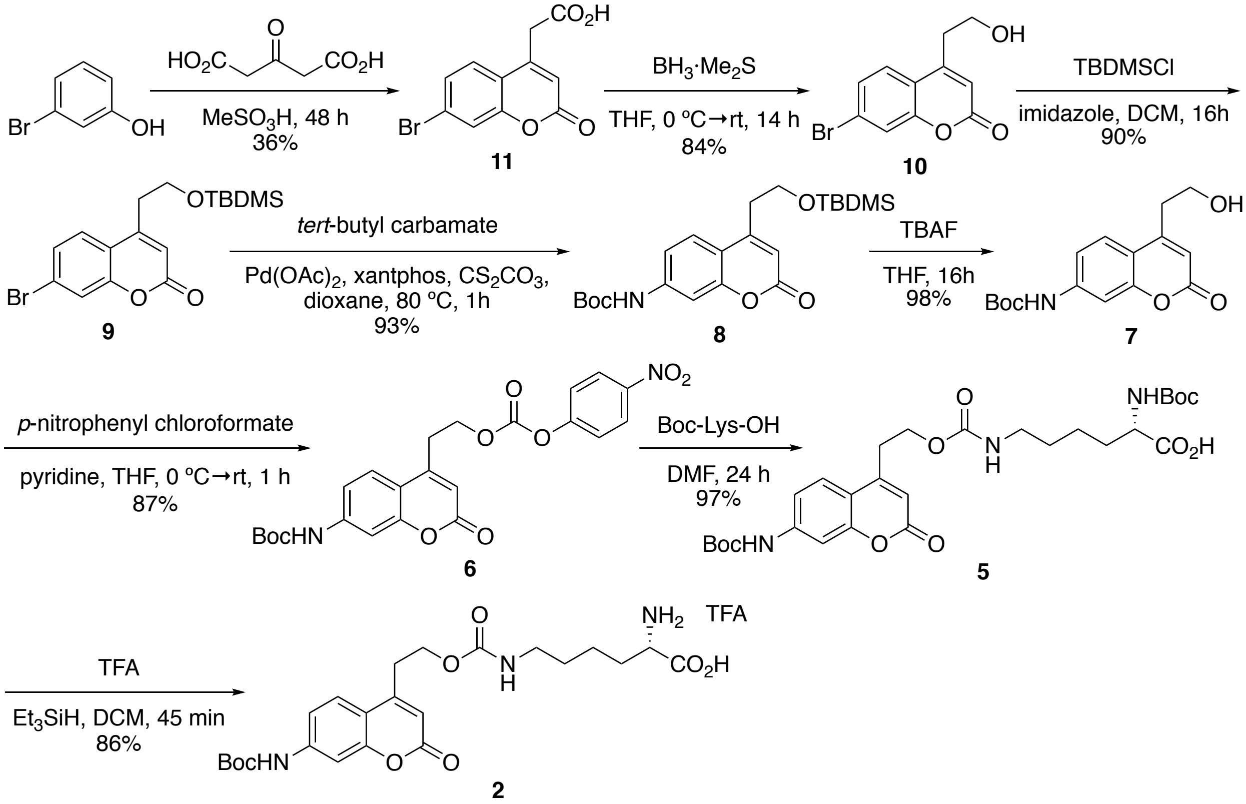

Scheme S2. Synthesis of the fluorescent 7-aminocoumarin lysine **2**.

2-(7-Bromo-2-oxo-2H-chromen-4-yl)acetic acid (11): Methanesulfonic acid (5 mL) was added to a mixture of 3-bromophenol (1.0 g, 5.78 mmol) and 3-oxopentanedioic acid (0.93 g, 6.36 mnol) and the resulting mixture was stirred at room temperature. After 70-80% consumption of the starting material over a period of 40-48 h, the reaction mixture was poured into ice-cold water with vigorous stirring. The resulting precipitate was washed with water and further purified by flash chromatography on silica gel to yield **11** (0.58 g, 36%) as white amorphous solid. ^{1}H NMR (300 MHz, acetone-*d*$_6$): δ 7.73 (d, *J* = 8.7 Hz, 1H), 7.60 (d, *J* = 1.8 Hz, 1H), 7.55 (dd, J_1 = 8.0 Hz, J_2 = 1.8 Hz, 1H), 6.53 (s, 1H), 3.99 (s, 2H).

7-Bromo-4-(2-hydroxyethyl)-2H-chromen-2-one (10): A 2 M solution of $BH_3{\cdot}Me_2S$ (265 µL, 0.53 mmol) in THF was added to an ice-cold solution of **11** (100 mg, 0.35 mmol) in anhydrous THF (2.0 mL), and the reaction mixture was stirred for 14 h and was allowed to warm to room temperature. The reaction was quenched with saturated Na_2CO_3 solution, concentrated and extracted with chloroform. The combined organic layers were washed with H_2O and brine, dried over anhydrous Na_2SO_4, filtered, and concentrated under vacuum. The residue was subsequently purified by flash chromatography on silica gel, eluting with 5% MeOH in $CHCl_3$ to afford **10** (80 mg, 84%) as white amorphous solid. ^{1}H NMR (300 MHz, acetone-*d*$_6$): δ 7.80 (d, *J* = 8.0 Hz, 1H), 7.56 (s, 1H), 7.51 (d, *J* = 8.0 Hz, 1H), 6.40 (s, 1H), 3.98 (brs, OH), 3.90-3.94 (m, 2H), 3.05 (t, *J* = 6.0 Hz, 2H); ^{13}C NMR (100 MHz, acetone-*d*$_6$): δ 159.9, 155.2, 164.3, 128.1, 127.6, 125.4, 120.6, 119.7, 116.2, 60.8, 34.2.

7-Bromo-4-(2-((*tert*-butyldimethylsilyl)oxy)ethyl)-2H-chromen-2-one (9): Imidazole (150 mg, 2.22 mmol) and *tert*-butyldimethylsilyl chloride (150 mg, 0.99 mmol) were added to a solution of alcohol **10** (150 mg, 0.56 mmol) in DCM (5 mL), and the resulting mixture was stirred at room temperature for 16 h. After quenching with water, the mixture was extracted with DCM. The combined DCM layers were washed with H_2O and brine, dried over anhydrous Na_2SO_4, filtered, and concentrated under vacuum. The residue was purified by flash chromatography on silica gel, eluting with 10% ethyl acetate in hexanes to give **9** (193 mg, 90%) as a white amorphous solid. ^{1}H NMR (400 MHz, $CDCl_3$): δ 7.55 (d, *J* = 8.4 Hz, 1H), 7.40 (d, *J* = 1.6 Hz, 1H), 7.37 (d, *J* = 8.4 Hz, 1H), 6.91 (s, 1H), 6.19 (s, 1H), 3.92 (t, *J* = 6.8 Hz, 2H), 2.93 (t, *J* = 6.8 Hz, 2H), 1.52 (s, 9H), 0.84 (s, 9H), -0.02 (s, 6H); ^{13}C NMR (100 MHz, $CDCl_3$): δ 161.3, 154.8, 153.6, 152.2, 142.1, 125.5, 114.7, 114.3, 113.1, 113.1, 105.9, 81.6, 61.7, 35.1, 28.4, 25.9, 18.3, -5.3.

***tert*-Butyl (4-(2-((*tert*-butyldimethylsilyl)oxy)ethyl)-2-oxo-2H-chromen-7-yl)carbamate (8)**: $Pd(OAc)_2$ (1.9 mg, 5 mol%), Cs_2CO_3 (85 mg, 2.6 mmol), xantphos (9.8 mg, 10 mol%), coumarin derivative **9** (65 mg, 0.17 mmol), and *tert*-butyl carbamate (25 mg, 0.21 mmol) were dissolved in anhydrous dioxane (1.0 mL) in an oven-dried, screw-capped pressure tube under an argon gas atmosphere. The reaction mixture was heated at 80 °C for 1 h while stirring. The reaction mixture was cooled to room temperature and the organic contents were extracted with ethyl acetate. The combined organic layers were washed with water and brine, dried over anhydrous Na_2SO_4, filtered, and concentrated under vacuum. The residue obtained was further purified by flash chromatography on silica gel, eluting with 20% ethyl acetate in hexanes to afford **8** (66 mg, 93%) as a white amorphous solid. ^{1}H NMR (300 MHz, $CDCl_3$): δ 7.53 (d, *J* = 8.1 Hz, 1H), 7.51 (s, 1H), 7.41 (dd, J_1 = 8.1 Hz, J_2 = 1.8 Hz, 1H), 6.34 (s, 1H), 3.94 (t, *J* = 6.3 Hz, 2H), 2.95 (t, *J* = 6.3 Hz, 2H), 0.84 (s, 9H), -0.01 (s, 6H); ^{13}C NMR (125 MHz, $CDCl_3$): δ 159.9, 153.9, 153.1, 127.4, 125.8, 125.4, 120.4, 118.5, 115.4, 61.4, 34.9, 25.7, 18.2, -5.5.

***tert*-Butyl (4-(2-hydroxyethyl)-2-oxo-2H-chromen-7-yl)carbamate (7):** A 1M solution of TBAF (1.1 mL, 0.29 mmol) was added to a solution of **8** (315 mg, 0.75 mmol) in THF (5.0 mL) and the resulting solution was stirred at room temperature for 16 h. The reaction mixture was quenched with saturated NH_4Cl solution, allowed to stir for 10 minutes, and extracted with DCM. The combined DCM layers were washed with H_2O and brine, dried over anhydrous Na_2SO_4, and

filtered. After removal of the solvent under vacuum, the residue was purified by flash column chromatography on silica gel to afford **7** (225 mg, 98%) as white solid. ^{1}H NMR (400 MHz, $CDCl_3$): δ 7.55 (d, *J* = 8.8 Hz, 1H), 7.38 (s, 1H), 7.36 (d, *J* = 8.8 Hz, 1H), 6.71 (s, 1H), 6.24 (s, 1H), 3.98-4.02 (m, 2H), 2.93 (t, *J* = 6.4 Hz, 2H), 1.61 (brs, OH), 1.56 (s, 9H); ^{13}C NMR (100 MHz, acetone-d_6): δ 160.9, 155.8, 154.6, 153.4, 144.0, 126.4, 115.0, 113.3, 105.9, 81.0, 61.0, 35.7, 28.4.

***tert*-Butyl (4-(2-(((4-nitrophenoxy)carbonyl)oxy)ethyl)-2-oxo-2H-chromen-7-yl)carbamate (6)**: Pyridine (80 µL, 0.99 mmol) was added dropwise to a mixture of *p*-nitrophenylchloroformate (198 mg, 0.98 mmol) and coumarin alcohol **7** (200 mg, 0.65 mmol) in anhydrous THF (5 mL) at 0 °C under an inert atmosphere. The reaction mixture was allowed to warm to room temperature and stirring was continued for 1 h. The reaction mixture was quenched with brine solution and was extracted with ethyl acetate. The combined organic layers were dried over anhydrous Na_2SO_4, filtrated, and concentrated under vacuum. The crude material was further purified by flash chromatography on silica gel, eluting with 10% ethyl acetate in DCM to yield coumarin carbonate **6** (265 mg, 87%) as a yellow solid. ^{1}H NMR (400 MHz, Acetone-d_6): δ 8.92 (brs, NH), 8.32 (d, *J* = 9.2 Hz, 2H), 7.81(d, *J* = 8.8 Hz, 1H), 7.69 (s, 1H), 7.51 (d, *J* = 8.8 Hz, 3H), 6.29 (s, 1H), 4.69 (t, *J* = 6.4 Hz, 2H), 3.33 (t, *J* = 6.4 Hz, 2H), 1.5 (s, 9H); ^{13}C NMR (100 MHz, Acetone-d_6): δ 160.7, 156.6, 155.8, 153.4, 153.1, 152.6, 146.5, 144.4, 126.2, 126.1, 123.1, 115.2, 114.5, 113.7, 106.0, 105.9, 81.1, 67.7, 31.2, 28.4.

***N^2*-(*tert*-Butoxycarbonyl)-*N^6*-((2-(7-((tert-butoxycarbonyl)amino)-2-oxo-2H-chromen-4-yl)ethoxy)carbonyl)-L-lysine (5):** Boc-L-lysine-OH (195 mg, 0.79 mmol) was added to a solution of coumarin carbonate **6** (272 mg, 0.58 mmol) in dry DMF (8 mL). The reaction mixture was stirred at room temperature under an inert gas atmosphere for 24 h. Then, the reaction mixture was poured into water (50 mL) and extracted using ethyl acetate. The combined organic layers were washed with water and brine, and dried over anhydrous Na_2SO_4. The filtrate was concentrated under vacuum and the resulting residue was purified by flash chromatography to obtain coumarin-lysine **5** (326 mg, 98%). ^{1}H NMR (500 MHz, CD_3OD): δ 7.72 (d, *J* = 8.8 Hz, 1H), 7.61 (s, 1H), 7.32 (d, *J* = 8.8 Hz, 1H), 6.22 (s, 1H), 4.34-4.37 (m, 2H), 3.85-3.98 (m, 1H), 3.05-3.13 (m, 4H), 1.71-1.82 (m, 1H), 1.50-1.69 (m, 1H), 1.54 (s, 9H), 1.26-1.49 (m, 13H); ^{13}C NMR (100 MHz, DMSO-d_6): δ 162.3, 160.0, 155.9, 155.0, 154.0, 153.1, 152.5, 143.2, 125.6, 114.3, 113.2, 111.9, 104.4, 79.9, 77.4, 61.5, 54.4, 48.6, 35.7, 30.8, 29.3, 28.2, 28.0, 22.6; HRMS (ESI+) calcd for $C_{28}H_{40}N_3O_{10}(M+H)^+$ 578.2714, found 578.2721.

***N^6*-((2-(7-Amino-2-oxo-2H-chromen-4-yl)ethoxy)carbonyl)-L-lysine** (**2**): A 1:1 mixture of TFA/DCM (3 mL) and triethylsilane (179 µL, 1.12 mmol) were added to **5** (325 mg, 0.56 mmol), and the mixture were stirred at room temperature for 45 min. Then, volatiles were removed under reduced pressure and the resulting oil was re-dissolved in MeOH (2 mL) and concentrated under reduced pressure. This process was repeated once more to remove residual TFA. Finally, the crude product was dissolved in minimum amount of MeOH (1 mL) and precipitated in Et_2O (100 mL) under vigorous stirring. The precipitate was collected and dried to furnish coumarin-lysine **2** (254 mg, 86%) as an amorphous white solid. ^{1}H NMR (500 MHz, CD_3OD): δ 7.53 (d, *J* = 8.4 Hz, 1H), 6.66 (d, *J* = 8.4 Hz, 1H), 6.52 (s, 1H), 5.98 (s, 1H), 4.32-4.36 (m, 2H), 3.71-3.73 (m, 1H), 3.06-3.09 (m, 4H), 1.82-1.92 (m, 2H), 1.42-1.50 (m, 4H); ^{13}C NMR (100 MHz, DMSO-d_6): δ 170.8, 160.6, 155.9, 155.7, 153.7, 153.1, 125.9, 111.3, 108.0, 107.5, 98.6, 61.8, 53.2, 48.5, 30.9, 30.3, 29.0, 22.0; HRMS (ESI+) calcd for $C_{18}H_{24}N_3O_6(M+H)^+$ 378.1665, found 378.1695.

Supporting Figures and Table

Table S1. Names and sequences of primers used in this study. Primers 1 and 2 amplified fLuc, primers 3 and 4 amplified rLuc, primers 5 and 6 amplified caPKA, primers 7 and 8 amplified NRAS, primers 9 and 10 make fLuc 529TAG, primers 11 and 12 make caPKA K72TAG, and primers 13 and 14 made NRAS G60E K16TAG. Primers 15 and 16 amplify axial for qPCR. Primers 17 and 18 amplify ef1a for qPCR.

**Primer**	**Sequence (5' to 3')**
1	ATAGAATACAAGCTACTTGTTCTTTTTGCAGGATCCATGGAAGACGCCAAAAACATAAAGAAAGGCCC
2	ACCACCGCCAGAACCGCCGCTACCGCCGCCCACGGCGATCTTTCCGCCCTTC
3	GGCGGCGGTAGCGGCGGTTCTGGCGGTGGTATGGCTTCCAAGGTGTACGACCCC
4	CACTATAGTTCTAGAGGCTCGAGAGGCCTTGAATTCTTACTGCTCGTTCTTCAGCACGCG
5	CATTAGGATCCATGGGCAACGCCGCCG
6	GATTAGAATTCTTAGAACTCGGTGAACTCCTTGCCGCAC
7	GAATAATCGATATGGCTTCTAGCTATCCTTATGACGTGCCTG
8	GATTAGAATTCTTACATCACCACACATGGCAATCCCATACAACC
9	CGAAAGGTCTTACCGGATAGCTCGACGCAAGAAAAATC
10	GATTTTTCTTGCGTCGAGCTATCCGGTAAGACCTTTCG
11	CGCCATGTAGATCCTGGACAAGCAGAAGGTG
12	GATCTACATGGCGTAGTGGTTGCCGCTCTC
13	GGGTAGAGCGCACTGACAATCCAGCTAATCC
14	GCGCTCTACCCAACACCACCTGCTCC
15	ATGCTCGGTGCTGTCAAA
16	CAAGTCCAGTGTTCATGTTGC
17	CTGGAGGCCAGCTCAAACAT
18	ATCAAGAAGAGTAGTACCGCTAGCATTAC

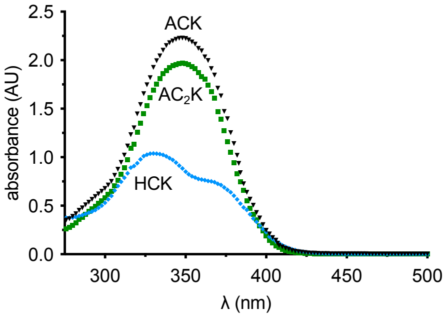

Figure S1. UV-Vis spectra of solutions of HCK, AC_2K, and ACK (0.2 mM in PBS containing 0.2% DMSO, pH 7.4). Absorbance from a blank (0.2% DMSO in PBS) was subtracted from each measurement. Spectra were recorded using a 1 cm quartz cuvette (FireflySci 30FLUV10 micro fluorescence cuvette) filled with 500 µL of each solution and were recorded using a Tecan M200 plate reader (2 nm step size, bandwidth 5 nm from 275-315 nm and 9 nm from 316-500 nm per default settings).

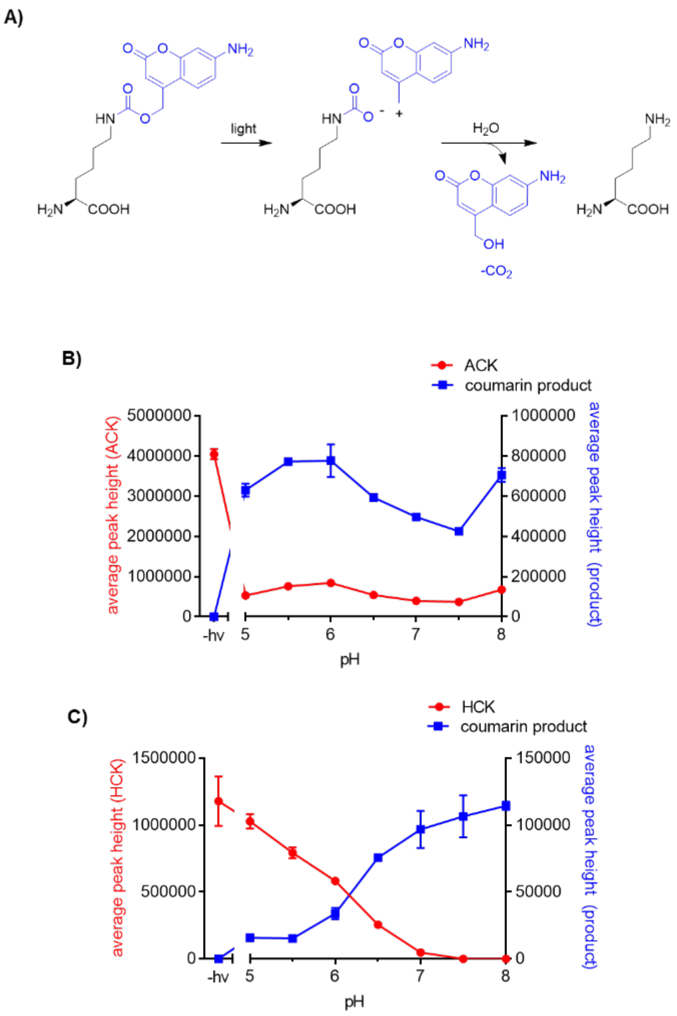

Figure S2. A) Decaging mechanism for ACK. Plot of LC-MS peak heights for B) ACK or C) HCK decaged with 405 nm light for 10 seconds at various pHs. The blue trace represents the coumarin caging group photoproduct and the red trace is the caged lysine. Data are the mean of duplicate samples and error bars are standard deviations.

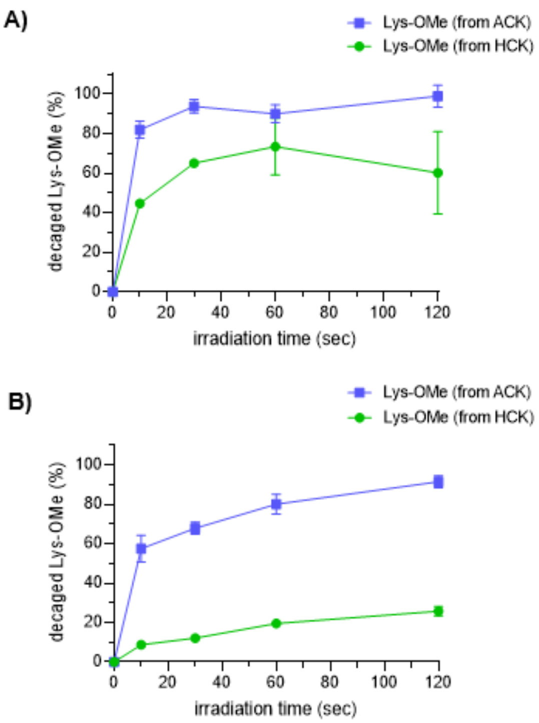

Figure S3. Comparison of released Lys-OMe from HCK-OMe and ACK-OMe through A) 365 nm and B) 405 nm irradiation. Irradiation was performed using LEDs and decaging reactions were analyzed by LC-MS. Data is the mean of duplicate experiments and error bars represent standard deviations.

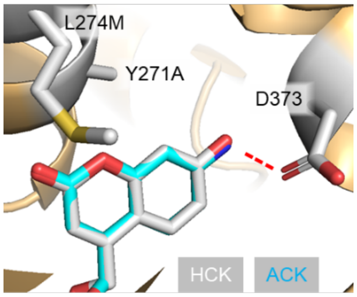

Figure S4. Docking of HCK (white) or ACK (cyan) into the HCKRS binding pocket (derived from PDB: 2Q7H). The HCKRS pocket mutations are shown. Hydrogen bonding is indicated with red dashes.

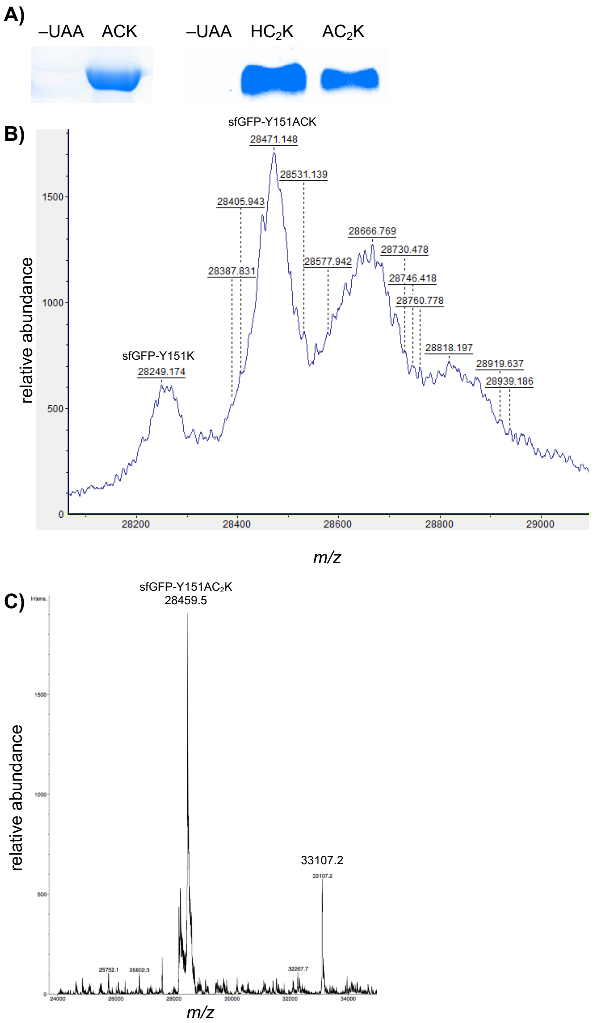

Figure S5. A) SDS-PAGE analysis of unnatural amino acid dependent sfGFP-Y151ACK and sfGFP-Y151AC$_2$K expression in *E. coli*. B) Whole-protein MALDI-TOF-MS analysis of sfGFP-Y151ACK. A small amount of decaged protein (sfGFP-Y151K) is noted, most likely due to decaging induced by the laser in the MALDI source (a frequency-tripled Bruker SmartBeam II Nd:YAG laser operating at 355 nm). Calcd [M+Na]$^+$: 28,467.915; found 28,471.148 (error: 3.233 Da or 114 ppm). sfGFP-Y151K: calcd [M+Na]$^+$: 28,250.736; found 28,249.174 (error: 1.562 Da or 55 ppm). C) Whole-protein ESI-MS analysis of sfGFP-Y151AC$_2$K. Calcd [M+H]$^+$: 28,459.9; found 28,459.5 (error: 0.4 Da or 14 ppm).

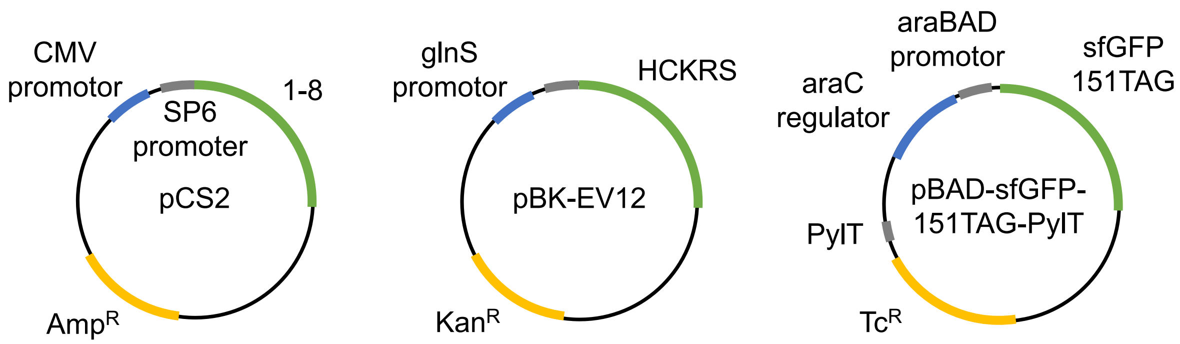

1. pCS2-HCKRS
2. pCS2-fLuc 529TAG-rLuc
3. pCS2-Cre
4. pCS2-Cre 201TAG
5. pCS2-caPKA
6. pCS2-caPKA 72TAG
7. pCS2-NRAS G60E
8. pCS2-NRAS G60E 16TAG

Figure S6. Maps of plasmids used in this study. The pCS2 vector is shown with the names of the constructs used in this study labelled 1-8 (shown as inserts downstream of the SP6 promoter).

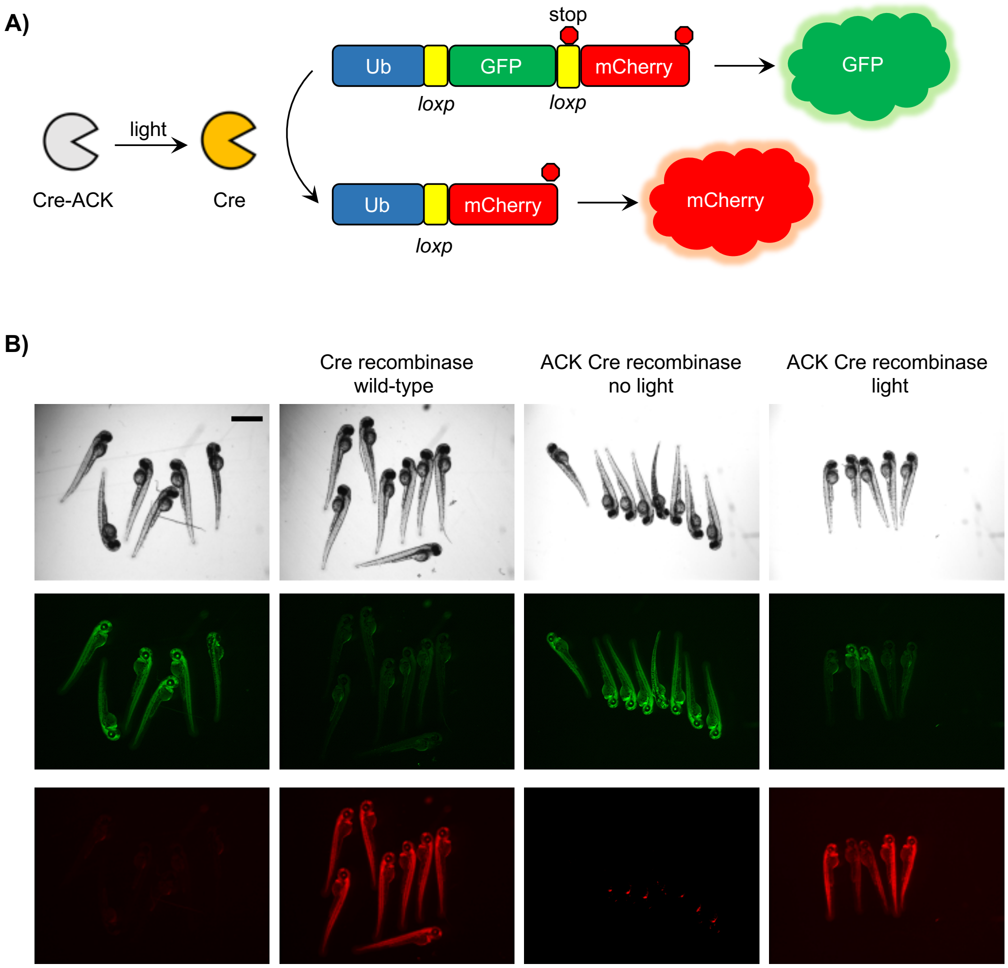

Figure S7. A) Recombination-induced switching from GFP to mCherry expression in response to irradiation of ACK-caged Cre recombinase (Cre-ACK). B) Micrographs showing photoactivation of ACK-caged Cre recombinase in Tg(ubi:loxp-EGFP-loxp-mCherry) embryos. Embryos were irradiated with a 405 nm LED (2 min) at 6 hpf and then imaged at 48 hpf. Scale bar = 1 mm.

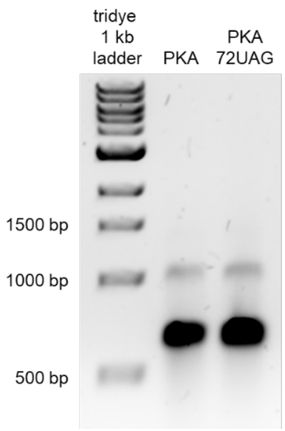

Figure S8. TBE-agarose gel (0.8%) electrophoresis of PKA mRNA (0.8%, 80 V, 45 min) stained with ethidium bromide (0.2 µg/mL included in TBE-agarose gel). A DNA ladder is included (TriDye 1 kb DNA Ladder, NEB).

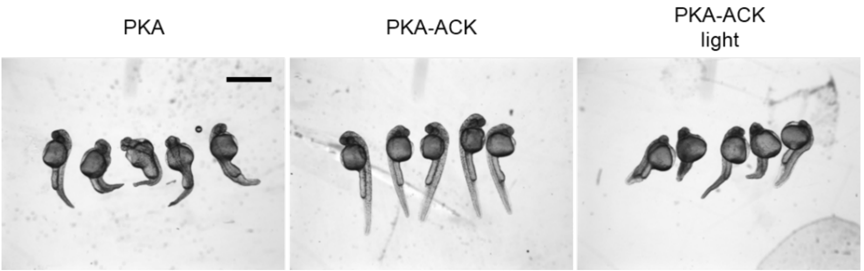

Figure S9. Representative batch images of caPKA photoactivation experiments (405 nm LED, 2 min). Scale bar = 1 mm.

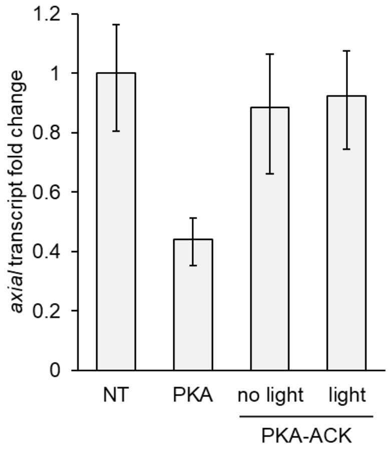

Figure S10. qPCR for axial transcripts was performed at 10 hpf with irradiation at 4 hpf with a 405 nm LED for 2 minutes. Bars represent mean and error bars represent standard deviation from triplicate total cDNA samples from pooled (50) embryos.

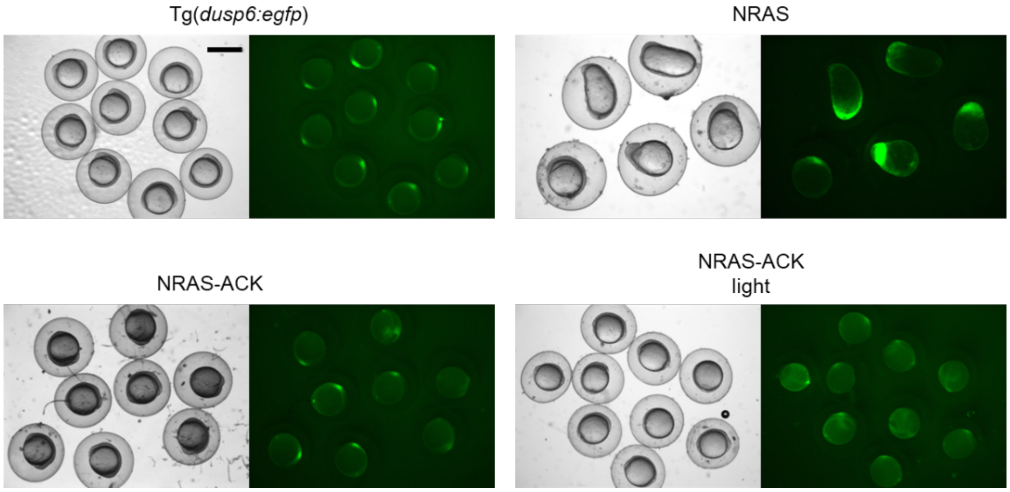

Figure S11. Batch images of Tg(*dusp6:egfp*) injected embryos irradiated at 6 hpf (405 nm LED, 2 min) and imaged at 10 hpf. Scale bar = 1 mm.

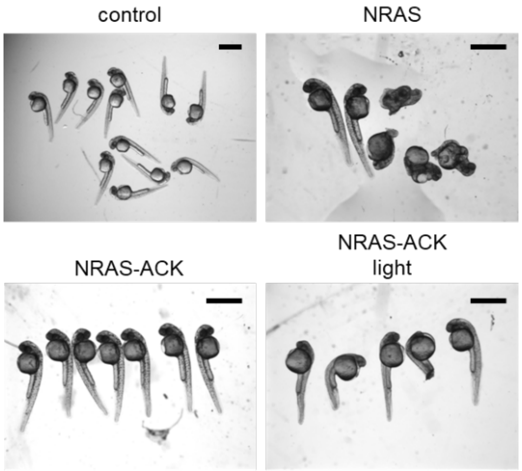

Figure S12. Representative batch images from NRAS photoactivation scoring experiments. Embryos were irradiated at 3 hpf (405 nm LED, 2 min) and imaged at 24 hpf. Scale bars = 1 mm.

Gene sequences

fLuc-rLuc (K529 boldface/underlined):

ATGGAAGACGCCAAAAACATAAAGAAAGGCCCGGCGCCATTCTATCCGCTGGAAGATGGAACCGCTGGAG
AGCAACTGCATAAGGCTATGAAGAGATACGCCCTGGTTCCTGGAACAATTGCTTTTACAGATGCACATAT
CGAGGTGGACATCACTTACGCTGAGTACTTCGAAATGTCCGTTCGGTTGGCAGAAGCTATGAAACGATAT
GGGCTGAATACAAATCACAGAATCGTCGTATGCAGTGAAAACTCTCTTCAATTCTTTATGCCGGTGTTGG
GCGCGTTATTTATCGGAGTTGCAGTTGCGCCCGCGAACGACATTTATAATGAACGTGAATTGCTCAACAG
TATGGGCATTTCGCAGCCTACCGTGGTGTTCGTTTCCAAAAAGGGGTTGCAAAAAATTTTGAACGTGCAA
AAAAAGCTCCCAATCATCCAAAAAATTATTATCATGGATTCTAAAACGGATTACCAGGGATTTCAGTCGA
TGTACACGTTCGTCACATCTCATCTACCTCCCGGTTTTAATGAATACGATTTTGTGCCAGAGTCCTTCGA
TAGGGACAAGACAATTGCACTGATCATGAACTCCTCTGGATCTACTGGTCTGCCTAAAGGTGTCGCTCTG
CCTCATAGAACTGCCTGCGTGAGATTCTCGCATGCCAGAGATCCTATTTTTGGCAATCAAATCATTCCGG
ATACTGCGATTTTAAGTGTTGTTCCATTCCATCACGGTTTTGGAATGTTTACTACACTCGGATATTTGAT
ATGTGGATTTCGAGTCGTCTTAATGTATAGATTTGAAGAAGAGCTGTTTCTGAGGAGCCTTCAGGATTAC
AAGATTCAAAGTGCGCTGCTGGTGCCAACCCTATTCTCCTTCTTCGCCAAAAGCACTCTGATTGACAAAT
ACGATTTATCTAATTTACACGAAATTGCTTCTGGTGGCGCTCCCCTCTCTAAGGAAGTCGGGGAAGCGGT
TGCCAAGAGGTTCCATCTGCCAGGTATCAGGCAAGGATATGGGCTCACTGAGACTACATCAGCTATTCTG
ATTACACCCGAGGGGGATGATAAACCGGGCGCGGTCGGTAAAGTTGTTCCATTTTTTGAAGCGAAGGTTG
TGGATCTGGATACCGGGAAAACGCTGGGCGTTAATCAAAGAGGCGAACTGTGTGTGAGAGGTCCTATGAT
TATGTCCGGTTATGTAAACAATCCGGAAGCGACCAACGCCTTGATTGACAAGGATGGATGGCTACATTCT
GGAGACATAGCTTACTGGGACGAAGACGAACACTTCTTCATCGTTGACCGCCTGAAGTCTCTGATTAAGT
ACAAAGGCTATCAGGTGGCTCCCGCTGAATTGGAATCCATCTTGCTCCAACACCCCAACATCTTCGACGC
AGGTGTCGCAGGTCTTCCCGACGATGACGCCGGTGAACTTCCCGCCGCCGTTGTTGTTTTGGAGCACGGA
AAGACGATGACGGAAAAAGAGATCGTGGATTACGTCGCCAGTCAAGTAACAACCGCGAAAAAGTTGCGCG
GAGGAGTTGTGTTTGTGGACGAAGTACCGAAAGGTCTTACCGGA**AAA**CTCGACGCAAGAAAAATCAGAGA
GATCCTCATAAAGGCCAAGAAGGGCGGAAAGATCGCCGTGGGCGGCGGTAGCGGCGGTTCTGGCGGTGGT
ATGGCTTCCAAGGTGTACGACCCCGAGCAACGCAAACGCATGATCACTGGGCCTCAGTGGTGGGCTCGCT
GCAAGCAAATGAACGTGCTGGACTCCTTCATCAACTACTATGATTCCGAGAAGCACGCCGAGAACGCCGT
GATTTTTCTGCATGGTAACGCTGCCTCCAGCTACCTGTGGAGGCACGTCGTGCCTCACATCGAGCCCGTG
GCTAGATGCATCATCCCTGATCTGATCGGAATGGGTAAGTCCGGCAAGAGCGGGAATGGCTCATATCGCC
TCCTGGATCACTACAAGTACCTCACCGCTTGGTTCGAGCTGCTGAACCTTCCAAAGAAAATCATCTTTGT
GGGCCACGACTGGGGGGCTTGTCTGGCCTTTCACTACTCCTACGAGCACCAAGACAAGATCAAGGCCATC
GTCCATGCTGAGAGTGTCGTGGACGTGATCGAGTCCTGGGACGAGTGGCCTGACATCGAGGAGGATATCG
CCCTGATCAAGAGCGAAGAGGGCGAGAAAATGGTGCTTGAGAATAACTTCTTCGTCGAGACCATGCTCCC
AAGCAAGATCATGCGGAAACTGGAGCCTGAGGAGTTCGCTGCCTACCTGGAGCCATTCAAGGAGAAGGGC
GAGGTTAGACGGCCTACCCTCTCCTGGCCTCGCGAGATCCCTCTCGTTAAGGGAGGCAAGCCCGACGTCG
TCCAGATTGTCCGCAACTACAACGCCTACCTTCGGGCCAGCGACGATCTGCCTAAGATGTTCATCGAGTC
CGACCCTGGGTTCTTTTCCAACGCTATTGTCGAGGGAGCTAAGAAGTTCCCTAACACCGAGTTCGTGAAG
GTGAAGGGCCTCCACTTCAGCCAGGAGGACGCTCCAGATGAAATGGGTAAGTACATCAAGAGCTTCGTGG
AGCGCGTGCTGAAGAACGAGCAGTAA

NLS SV40-Cre recombinase (K201 boldface/underlined):

ATGGCTCCAAAGAAGAAGCGTAAGGTATCCAATTTACTGACCGTACACCAAAATTTGCCTGCATTACCGG
TCGATGCAACGAGTGATGAGGTTCGCAAGAACCTGATGGACATGTTCAGGGATCGCCAGGCGTTTTCTGA
GCATACCTGGAAAATGCTTCTGTCCGTTTGCCGGTCGTGGGCGGCATGGTGCAAGTTGAATAACCGGAAA
TGGTTTCCCGCAGAACCTGAAGATGTTCGCGATTATCTTCTATATCTTCAGGCGCGCGGTCTGGCAGTAA
AAACTATCCAGCAACATTTGGGCCAGCTAAACATGCTTCATCGTCGGTCCGGGCTGCCACGACCAAGTGA
CAGCAATGCTGTTTCACTGGTTATGCGGCGGATCCGAAAAGAAAACGTTGATGCCGGTGAACGTGCAAAA
CAGGCTCTAGCGTTCGAACGCACTGATTTCGACCAGGTTCGTTCACTCATGGAAAATAGCGATCGCTGCC
AGGATATACGTAATCTGGCATTTCTGGGGATTGCTTATAACACCCTGTTACGTATAGCCGAAATTGCCAG
GATCAGGGTTAAAGATATCTCACGTACTGACGGTGGGAGAATGTTAATCCATATTGGCAGAACG**<u>AAA</u>**ACG
CTGGTTAGCACCGCAGGTGTAGAGAAGGCACTTAGCCTGGGGGTAACTAAACTGGTCGAGCGATGGATTT
CCGTCTCTGGTGTAGCTGATGATCCGAATAACTACCTGTTTTGCCGGGTCAGAAAAAATGGTGTTGCCGC
GCCATCTGCCACCAGCCAGCTATCAACTCGCGCCCTGGAAGGGATTTTTGAAGCAACTCATCGATTGATT
TACGGCGCTAAGGATGACTCTGGTCAGAGATACCTGGCCTGGTCTGGACACAGTGCCCGTGTCGGAGCCG
CGCGAGATATGGCCCGCGCTGGAGTTTCAATACCGGAGATCATGCAAGCTGGTGGCTGGACCAATGTAAA
TATTGTCATGAACTATATCCGTAACCTGGATAGTGAAACAGGGGCAATGGTGCGCCTGCTGGAAGATGGC
GATCTCGAGTAA

caPKA (K72 boldface/underlined):

ATGGGCAACGCCGCCGCCGCCAAGAAGGGCAGCGAGCAGGAGAGCGTGAAGGAGTTCCTGGCCAAGGCCA
AGGAGGACTTCCTGAAGAAGTGGGAGACCCCCAGCCAGAACACCGCCCAGCTGGACCAGTTCGACAGAAT
CAAGACCCTGGGCACCGGCAGCTTCGGCAGAGTGATGCTGGTGAAGCACAAGGAGAGCGGCAACCACTAC
GCCATG**<u>AAG</u>**ATCCTGGACAAGCAGAAGGTGGTGAAGCTGAAGCAGATCGAGCAGACCCTGAACGAGAAGA
GAATCCTGCAGGCCGTGAACTTCCCCTTCCTGGTGAAGCTGGAGTTCAGCTTCAAGGACAACAGCAACCT
GTACATGGTGATGGAGTACGTGGCCGGCGGCGAGATGTTCAGCCACCTGAGAAGAATCGGCAGATTCAGC
GAGCCCCACGCCAGATTCTACGCCGCCCAGATCGTGCTGACCTTCGAGTACCTGCACAGCCTGGACCTGA
TCTACAGAGACCTGAAGCCCGAGAACCTGCTGATCGACCAGCAGGGCTACATCCAGGTGACCGACTTCGG
CTTCGCCAAGAGAGTGAAGGGCAGAACCAGAACCCTGTGCGGCACCCCCGAGTACCTGGCCCCCGAGATC
ATCCTGAGCAAGGGCTACAACAAGGCCGTGGACTGGTGGGCCCTGGGCGTGCTGATCTACGAGATGGCCG
CCGGCTACCCCCCCTTCTTCGCCGACCAGCCCATCCAGATCTACGAGAAGATCGTGAGCGGCAAGGTGAG
ATTCCCCAGCCACTTCAGCAGCGACCTGAAGGACCTGCTGAGAAACCTGCTGCAGGTGGACCTGACCAAG
AGATTCGGCAACCTGAAGAACGGCGTGAACGACATCAAGAACCACAAGTGGTTCGCCACCACCGACTGGA
TCGCCATCTACCAGAGAAAGGTGGAGGCCCCCTTCATCCCCAAGTTCAAGGGCCCCGGCGACACCAGCAA
CTTCGACGACTACGAGGAGGAGGAGATCAGAGTGAGCATCAACGAGAAGTGCGGCAAGGAGTTCACCGAG
TTCTAA

NRAS G60E-HA (K16 boldface/underlined):

ATGGCTTCTAGCTATCCTTATGACGTGCCTGACTATGCCAGCCTGGGAGGACCTTCTAGCGGATCCACCA
TGACTGAGTAC**<u>AAA</u>**CTGGTGGTGGTTGGAGCAGGTGGTGTTGGGAAAAGCGCACTGACAATCCAGCTAAT
CCAGAACCACTTTGTAGATGAATATGATCCCACCATAGAGGATTCTTACAGAAAACAAGTGGTTATAGAT
GGTGAAACCTGTTTGTTGGACATACTGGATACAGCTGAGCAAGAAGAGTACAGTGCCATGAGAGACCAAT
ACATGAGGACAGGCGAAGGCTTCCTCTGTGTATTTGCCATCAATAATAGCAAGTCATTTGCGGATATTAA
CCTCTACAGGGAGCAGATTAAGCGAGTAAAAGACTCGGATGATGTACCTATGGTGCTAGTGGGAAACAAG
TGTGATTTGCCAACAAGGACAGTTGATACAAAACAAGCCCACGAACTGGCCAAGAGTTACGGGATTCCAT
TCATTGAAACCTCAGCCAAGACCAGACAGGGTGTTGAAGATGCTTTTTACACACTGGTAAGAGAAATACG
CCAGTACCGAATGAAAAAACTCAACAGCAGTGATGATGGGACTCAGGGTTGTATGGGATTGCCATGTGTG
GTGATGTAA

Supporting References

1. Gibson, D. G.; Young, L.; Chuang, R.-Y.; Venter, J. C.; Hutchison, C. A.; Smith, H. O., Enzymatic assembly of DNA molecules up to several hundred kilobases. *Nat. Methods* **2009,** *6* (5), 343-345.
2. Luo, J.; Uprety, R.; Naro, Y.; Chou, C.; Nguyen, D. P.; Chin, J. W.; Deiters, A., Genetically encoded optochemical probes for simultaneous fluorescence reporting and light activation of protein function with two-photon excitation. *J. Am. Chem. Soc.* **2014,** *136* (44), 15551-8.
3. Uprety, R.; Luo, J.; Liu, J.; Naro, Y.; Samanta, S.; Deiters, A., Genetic Encoding of Caged Cysteine and Caged Homocysteine in Bacterial and Mammalian Cells. *ChemBioChem* **2014,** *15* (12), 1793-1799.
4. Wesalo, J. S.; Luo, J.; Morihiro, K.; Liu, J.; Deiters, A., Phosphine-Activated Lysine Analogues for Fast Chemical Control of Protein Subcellular Localization and Protein SUMOylation. *ChemBioChem* **2020,** *21* (1-2), 141-148.
5. Cürten, B.; Kullmann, P. H. M.; Bier, M. E.; Kandler, K.; Schmidt, B. F., Synthesis, Photophysical, Photochemical and Biological Properties of Caged GABA, 4-[[(2H-1-Benzopyran-2-one-7-amino-4-methoxy) carbonyl] amino] Butanoic Acid. *Photochem. Photobiol.* **2005,** *81* (3), 641-648.
